# The Emergence of Resistance to the Benzimidazole Anthlemintics in Parasitic Nematodes of Livestock Is Characterised by Multiple Independent Hard and Soft Selective Sweeps

**DOI:** 10.1371/journal.pntd.0003494

**Published:** 2015-02-06

**Authors:** Elizabeth Redman, Fiona Whitelaw, Andrew Tait, Charlotte Burgess, Yvonne Bartley, Philip John Skuce, Frank Jackson, John Stuart Gilleard

**Affiliations:** 1 Department of Comparative Biology and Experimental Medicine, Faculty of Veterinary Medicine, University of Calgary, Calgary, Alberta, Canada; 2 School of Veterinary Medicine, College of Medical, Veterinary and Life Sciences, University of Glasgow, Glasgow, United Kingdom; 3 Moredun Research Institute, Pentlands Science Park, Midlothian, United Kingdom; University of Pennsylvania, UNITED STATES

## Abstract

Anthelmintic resistance is a major problem for the control of parasitic nematodes of livestock and of growing concern for human parasite control. However, there is little understanding of how resistance arises and spreads or of the “genetic signature” of selection for this group of important pathogens. We have investigated these questions in the system for which anthelmintic resistance is most advanced; benzimidazole resistance in the sheep parasites *Haemonchus contortus* and *Teladorsagia circumcincta*. Population genetic analysis with neutral microsatellite markers reveals that *T. circumcincta* has higher genetic diversity but lower genetic differentiation between farms than *H. contortus* in the UK. We propose that this is due to epidemiological differences between the two parasites resulting in greater seasonal bottlenecking of *H. contortus*. There is a remarkably high level of resistance haplotype diversity in both parasites compared with drug resistance studies in other eukaryotic systems. Our analysis suggests a minimum of four independent origins of resistance mutations on just seven farms for *H. contortus*, and even more for *T. circumincta*. Both hard and soft selective sweeps have occurred with striking differences between individual farms. The sweeps are generally softer for *T. circumcincta* than *H. contortus*, consistent with its higher level of genetic diversity and consequent greater availability of new mutations. We propose a model in which multiple independent resistance mutations recurrently arise and spread by migration to explain the widespread occurrence of resistance in these parasites. Finally, in spite of the complex haplotypic diversity, we show that selection can be detected at the target locus using simple measures of genetic diversity and departures from neutrality. This work has important implications for the application of genome-wide approaches to identify new anthelmintic resistance loci and the likelihood of anthelmintic resistance emerging as selection pressure is increased in human soil-transmitted nematodes by community wide treatment programs.

## Author Summary

Parasitic nematodes (roundworms) are major causes of disease in both domestic animals and humans. Strategic treatments with anthelmintic drugs have been used to control livestock parasites for several decades resulting in widespread drug resistance. Drug treatments have, until recently, been applied at a relatively low level to control human parasites. However, in recent years community wide treatment programs have been massively increased for the 1–2 billion people infected with roundworms. Hence, for both human and animal health, there is an urgent need to understand how resistance emerges and spreads and how we can detect resistance mutations in this important group of pathogens. In this study, we investigated how drug resistance mutations appear and spread in the two livestock parasites for which resistance is most widespread. We have found that resistance appears repeatedly and frequently in parasite populations, and propose a model to explain the high capacity of these pathogens to develop drug resistance. Our work suggests that anthelmintic resistance is likely to occur when repeated drug treatment is relied upon to control this group of pathogens. Our results also suggest that resistance mutations should be detectable when modern genome-wide approaches are used to scan the genomes of resistant parasites.

## Introduction

Understanding the processes that affect the emergence of drug resistance in eukaryotic pathogens is an important goal. Anthelmintic resistance in parasitic nematodes is a threat to sustainable livestock production worldwide and a growing concern for the control of human parasites in the developing world [1]. Our limited understanding of how resistance mutations arise and spread in parasitic nematode populations limits our ability to develop evidence-based mitigation strategies. Moreover, there is little information on the changes that occur in the genome as anthelmintic resistance mutations increase in frequency in parasite populations; the so called “genetic signature” of selection. Such knowledge is critical if we are to apply genome-wide population genomic approaches to identify new anthelmintic resistance mutations.

*Haemonchus contortus* and *Teladorsagia circumcincta* are two closely related parasitic nematodes of sheep for which anthelmintic resistance is widespread. In these parasites, resistance has occurred against all broad spectrum anthelmintic classes used in their control [1]. Resistance to benzimidazoles is particularly common due to their intensive use since the 1970s and is at least partially understood at the molecular level. Several mutations in the isotype-1 β-tubulin gene, which encodes the drug target, have been shown to be associated with resistance. Substitutions of a phenylalanine for a tyrosine at codon positions 167 and 200 (F167Y and F200Y) of the isotype-1 β-tubulin polypeptide have been reported in both species and a substitution of glutamic acid for alanine at position 198 (E198A) has also been described in *H*. *contortus* [2–9]. All three of these substitutions have been shown to be associated with the resistance phenotype and the F200Y substitution has been subject to extensive functional analysis [6,10]. Consequently, benzimidazole resistance in these two parasite species is currently the best system in which to study the population genetics of anthelmintic resistance in parasitic nematodes.

In this study, we have investigated the population structure of *T*. *circumcincta* and *H*. *contortus* and studied the genetics of benzimidazole resistance on seven commercial sheep farms in the UK. The two species were sampled from the same animals on each farm to allow a direct comparison without any confounding differences in environment or management. The aim was to investigate how anthelmintic resistance mutations emerge and spread in response to selection. In the classical model of adaptation, a beneficial mutation arises once and increases in frequency under the influence of selection. This is known as a “hard” selective sweep and is characterised by a single haplotype increasing in frequency. There is a dramatic loss of marker polymorphism around the selected locus which is known as the “hitchhiking effect” [11]. However, it is also possible for multiple adaptive alleles to sweep through a population, particularly when there is rapid adaptation, and this leads to a more complex genetic signature of selection [12]. This is known as a “soft” selective sweep in which multiple haplotypes originate either from the standing genetic variation or arise independently by recurrent *de novo* mutations. In this study, we report a remarkably high diversity of benzimidazole resistance haplotypes with both hard and soft selective sweeps occurring in different parasite populations. We propose a model for anthelmintic resistance in which multiple independent resistance mutations recurrently arise in parasite populations and are then spread by migration. This explains why anthelmintic resistance is so common in livestock parasites and suggests that the emergence of resistance is likely whenever parasitic nematodes with large population sizes are exposed to intensive drug selection.

## Materials and Methods

### Identification of the study farms

**Fig 1:**
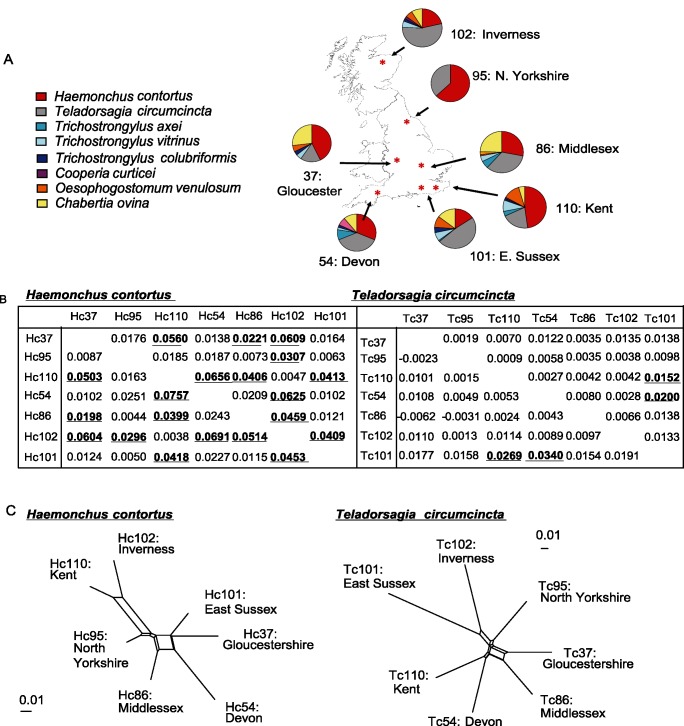
Prevalence of parasitic nematode species identified on the seven study farms and parasite population structure. (A) The relative frequencies of the eight major ovine gastro-intestinal nematode species based on species-specific PCR assays on 90 individual L_1_ larvae on the seven study farms. (B) Pairwise F_ST_ values based on genotyping with ten microsatellite loci for *H*. *contortus* and *T*. *circumcincta* respectively. F_ST_ values estimated before correction for null alleles below the diagonal and after correction above. Values underlined and in bold show significant differences based on bootstrapping (10, 000 replicates) using the software program FreeNA. (C) SplitsTrees4 Networks based on populations of thirty worms on each farm genotyped using ten microsatellite markers for each species.

In order to enable a direct comparison of the population genetics of *T*. *circumcincta* and *H*. *contortus*, farms with high levels of infection of both species first had to be identified. As part of a previously published survey of 91 UK sheep farms sampled in 2008, we had identified 45 farms on which both *T*. *circumcincta* and *H*. *contortus* worms were present in ewes at lambing [13]. Eggs were extracted from a pool of faeces from 20 ewes per farm and allowed to hatch into L_1_ larvae. DNA templates were made from approximately 1000 pooled L_1_ larvae from each of the 45 farms and then screened with three species-specific microsatellite markers (*H*. *contortus*; Hcms25, Hcms53265 and Hcms22193; *T*. *circumcincta* loci, mtg67, mtg68 and mtg73) to identify farms on which both species were present at high frequency (based on the identification of numerous alleles). The prevalence of the two species was then more accurately established on 20 chosen farms using single worm rDNA ITS-2 species-specific PCR assays on 90 individual L_1_ per farm (S1 Fig.). Seven farms with a high prevalence of both *H*. *contortus* and *T*. *circumcincta* infection were chosen for detailed population genetic analysis: farm 37 (Gloucester), farm 54 (Devon), farm 86 (Middlesex), farm 95 (North Yorkshire), farm 101 (East Sussex), farm 102 (Inverness) and farm 110 (Kent) (Fig. 1A). The prevalence of the other major ovine gastrointestinal nematode species on these seven study farms was determined by applying rDNA ITS-2 species-specific PCR assays to 90 L_1_ individual larvae for *H*. *contortus* [14], *Trichostrongylus axei*, *Trichostrongylus vitrinus*, [15] *T*. *circumcincta*, *Trichostrongylus colubriformis*, *Cooperia curticei* [13], *Chabertia ovina* [15] and *Oesophagostomum venulosum* (assay developed in this study, S1 Table). These data, along with the location of the farms are shown in Fig. 1A. 30 individual L_1_ DNA lysates for *T*. *circumcincta* and *H*. *contortus* were then used for the detailed population analysis undertaken in this study.

### PCR methodology

Bulk DNA lysates were made using previously described techniques [14] and 1l of a 1:40 dilution of neat lysate was used as PCR template. Dilutions of several aliquots of lysate buffer, made in parallel, were included as negative controls for all PCR amplifications. Conditions for the rDNA ITS-2 species-specific PCR assays were 94°C for 2 min followed by 35 cycles of 92°C for 30s (the exception being *H*. *contortus* with 40 cycles); annealing temperature for 30s and 72°C for 30s with a single final extension cycle at 72°C for 10 min. PCR primers and annealing temperatures are given in S1 Table.

### Microsatellite identification, development and amplification

A number of microsatellite loci have previously been characterised for *H*. *contortus* [14,16,17] and *Teladorsagia circumcincta* [18]. In order to identify additional loci for this study, Tandem Repeats Finder [19] was used to search the 2006 *H*. *contortus* and the 2009 *T*. *circumcincta* supercontig databases (ftp.sanger.ac.uk/pub/pathogens/). Loci chosen on the basis of repeat purity and sufficient flanking sequence were tested for consistency of amplification and allelic polymorphism using a range of genetically divergent isolates (*H*. *contortus*: MHco3(ISE), Hco4(WRS), Hco10(CAVR) and a 2007 UK ovine field isolate designated Hco(UK-12/10/07) [14]; *T*. *circumcincta*: MTci5, NzWS, FrGa and ScSo210 [20]). A summary of the screening results is shown in S2 Table and the primer sequences, repeat motifs and allele ranges of the new loci are given in S3 Table. A final panel of ten microsatellite loci were chosen as population markers for this study for each species: *T*. *circumcincta* (previously described loci: mtg15, mtg67, mtg68, mtg73; new loci: Tc22274, Tc7989, Tc4504, Tc2066, Tc2467, Tc13604) and a panel of ten *H*. *contortus* loci (previously described loci: Hcms25, Hcms36, Hcms40, Hcms22co3; new loci: Hc53265, Hc2884, Hc3086, Hc22193, Hc12850, Hc13507).

The PCR conditions for microsatellite amplification have been previously described [14] [18]. The forward primer of each microsatellite primer pair was 5’-end labelled with FAM, HEX, or NED fluorescent dyes and amplicons were electrophoresed, with a GeneScan ROX 400 (Applied Biosystems) internal size standard, on an ABI Prism 3100 Genetic Analyzer (Applied Biosystems). Individual chromatograms were analyzed using Genemapper Software Version 4.0 (Applied Biosystems).

### Population genetic analysis

From multi-locus microsatellite genotype data, heterozygosity (H_e_ and H_o_), allele richness and estimates of F_IS_ for each locus were calculated by Arlequin 3.1 [21]. Guo & Thompson’s (1992) Exact Test was used to statistically evaluate deviations from Hardy—Weinberg equilibrium for all populations [22]. Significance levels were adjusted using the sequential method of Bonferroni for multiple comparisons in the same dataset [23]. Linkage disequilibrium analysis was carried out by GENEPOP version 3.3 [24] using a log likelihood ratio test statistic (G-test). Partition of microsatellite diversity between and within farm populations was estimated through an analysis of molecular variance AMOVA [25]. Data were defined as ‘standard’ rather than ‘microsatellite’, as loci did not necessarily adhere to the stepwise mutation model. Pairwise F_ST_ values were calculated and significance testing was undertaken by random permutation in Arlequin 3.11. Tests for the presence and influence of null alleles were conducted using FreeNA [26], a software program that is able to produce F_ST_ estimates before and after a correction for null alleles and utilizes bootstrapping (with 10,000 replicates) to determine significance.

### Pyrosequence genotyping of benzimidazole resistance-associated SNPs in the isotype-1 β-tubulin locus

Non-synonymous single nucleotide polymorphisms (SNPs) in the isotype-1 β-tubulin gene have been previously associated with benzimidazole resistance in *H*. *contortus* at codons P167, P198, and P200 [4–7,27] and at codons P167 and P200 for *T*. *circumcincta* [2,3,7–9]. Pyrosequencing assays designed to target the F167Y (T**T**C>T**A**C), F200Y (T**T**C>T**A**C) and E198A (G**A**A>G**C**A) SNPs of the isotype-1 β-tubulin genes of *H*. *contortus* and *T*. *circumcincta* were used to estimate the prevalence of each resistant mutation on each farm. Genotypes were generated for 30 single worms per farm population. These assays have been described elsewhere: *T. circumcincta [9]* and *H*. *contortus* [28].

### Sequencing of isotype-1 β-tubulin haplotypes

A fragment encompassing the F167Y (T**T**C>T**A**C), F200Y (T**T**C>T**A**C) and E198A (G**A**A>G**C**A) SNPs in the isotype-1 β-tubulin gene was PCR amplified from the same individual worms that were genotyped. Specific primers anchored in exon 3 and 7 (Tc37F: GCTGAGCTTGTTGACAACG

Tc37R AGATAGCGTCCGTGGCGAG; Hc37F GCCGAGCTAGTTGATAACG

Hc37R AGATAACGTCCATGGCGAG) were used to amplify a 922bp fragment for *H*. *contortus* and a 940bp fragment for *T*. *circumcincta*. The PCR conditions were the same for both species (95°C for 5min followed by 45 cycles of 94°C for 15s; 60°C for 1min and 72°C for 1min with a single final extension cycle at 72°C for 20min). HotStar HiFidelity Polymerase (Qiagen) was used with the proprietary Q-solution to minimize the number of polymerase-induced errors. Amplicons from the 30 individual worms per farm population were pooled together and cloned, using the Zero Blunt cloning vector, Invitrogen (7 cloning reactions per species). A minimum of 20 clones were selected and sequenced per cloning reaction to provide adequate representation of the most common haplotypes on each farm. Each clone was sequenced in both orientations to create a consensus sequence for each haplotype.

### Estimation of PCR error rate

The error rate (R) for the PCR amplification of the isotype-1 β-tubulin was experimentally determined. A 922bp region of the *H*. *contortus* and a 940bp region of the *T*. *circumcincta* isotype-1 β-tubulin target was independently PCR amplified on 10 separate occasions from a single worm of each species that was heterozygous for the P200 polymorphism. Each amplicon was cloned and a single clone of each allele sequenced in both orientations. This approach yielded 10 sequences each corresponding to one of the two different alleles present in a single diploid worm for each species. The error rate (R) per bp per cycle was calculated by dividing the total number of sequence polymorphisms by the total length of sequence, L (when L = lcn and l = length of amplicon, c = number of cycles, n = number of PCR reactions). For example, from the 10 sequences of the resistant (P200Y) *H*. *contortus* allele a total of 3 polymorphisms were identified. This equated to an error rate of 7.23*10^–6^ (3/ 922*45*10). The error rates calculated for *H*. *contortus* and *T*. *circumcincta* using this method were similar to each other (*T*. *circumcinta*: 7.09–9.46*10^–6^, *H*. *contortus*: 7.23–9.64*10^–6^). The error rate (R) can then be used to estimate the fraction of PCR-induced mismatches (F) with the formula 1-e^-lRc^ [29]. For *H*. *contortus* and *T*. *circumcincta* the fraction of PCR-induced mismatches (F) proved to be identical to 3 decimal places, (F = 0.259–0.330, 1-e^-922*(7.23*10–6)*45^ = 0.259 and 1-e^-922*(9.64*10–6)*45^ = 0.330), suggesting that approximately one in every three or four, isotype-1 β-tubulin sequences would contain a PCR-induced mutation.

### Bioinformatic filtering of isotype-1 β-tubulin sequences to remove PCR induced mutations from the dataset

SNPs appearing more than once in a sequence data set of the size analyzed in this work (140 sequences for each species) are highly likely to be real polymorphisms, whereas SNPs that only occur once are possible artifacts due to polymerase induced errors. In order to filter our dataset, the frequency distribution of the SNPs was plotted along the isotype-1 β-tubulin gene model (S2 Fig.). The SNPs were classified into two groups: those that occurred only once and those that occurred more than once in the 140 sequence dataset. The distribution patterns for these two SNP categories strongly support the hypothesis that most, if not all, “unique” SNPs were PCR-induced mutations whereas those appearing more than once were genuine polymorphisms. “Unique” SNPs were evenly distributed across introns and exons and there was no bias for synonymous versus non-synonymous mutations for the exonic SNPs (S2 Fig.). In contrast, SNPs appearing more than once in the dataset were clustered within the introns and, for the exonic SNPs, there was bias to synonymous changes. The only non-synonymous SNPs appearing more than once in the dataset occurred at the resistance associated positions P167, P198 and P200 (S2 Fig.). In order to take a conservative approach and ensure only real polymorphisms were considered in our analyses, all SNPs occurring only once in the dataset were discarded. This resulted in 15 different *H*. *contortus* and 43 different *T*. *circumcincta* haplotypes represented in this final “filtered” sequence data used for all subsequent analysis.

### Phylogenetic network analysis

Haplotype networks were generated from the isotype-1 β-tubulin sequence data based on genetic distance with the SplitsTree4 program [30]. Initially a distance matrix based on the proportion of positions at which any two isotype-1 β-tubulin sequences differed, was created (Uncorrected-P option). Circular (equal angle) split networks were generated from the distance matrices with the neighbour-net method. Neighbour-net networks were also constructed from microsatellite data from each farm. Each split in a network between any two individuals or farms is displayed by parallel edges whose length is proportional to the weight of the associated split. Networks were also generated with the software TCS [31]. This is based on the concept of statistical parsimony for which all pairs of haplotypes are compared and the connections or “edges” between the haplotypes are scored according to their “probability of parsimony”. Only edges with a probability of parsimony of greater than 95% are used for the construction of the haplotype network. Analysis was conducted on a multiple alignment of “collapsed” sequences where allele frequency information had been removed. Gaps in sequences were classified as “missing” data. The analysis was able to infer the most probable “ancestral” haplotypes. Each branch represents a single nucleotide mutation and empty nodes are assumed haplotypes. Statistical support was generated through the calculation of consistency indicies (CI) in Winclada [32].

In addition, Median Joining networks were generated in Network 4.6.1 (Fluxus Technology Ltd.). Sequences were initially aligned in ClustalX and prepared for import using DNA Alignment software (Fluxus Technology Ltd). A full median network containing all possible shortest trees was generated by setting the epsilon parameter equal to the greatest weighted distance (epsilon = 10). All unnecessary median vectors and links were removed with the MP (Maximum Parsimony) option [33]. Small black dots represent median vectors and the number of mutations separating adjacent sequence nodes and/or median vectors are indicated along connecting branches. The most probable ancestral node was determined by rooting the network to a closely related outgroup; a *T*. *circumcincta* sequence was used to root the *H*. *contortus* network and vice versa.

### Sequence diversity estimates, tests for selective neutrality and recombination analysis

The following diversity indices were calculated using DnaSP 5.10 [34]: nucleotide diversity (π), Gene diversity (H_d_), the mean number of pairwise differences (k), the number of segregating sites (S); and the Mutation parameter based on an infinite site equilibrium model and the number of segregating sites (θ_S_). Tests for selective neutrality were analysed with the program DnaSP 5.10 to determine whether the observed frequency distribution of nucleotide polymorphism departs from neutral expectations. Neutrality tests were conducted included Tajima’s *D* [35], and Fay and Wu’s *H* [36]. These were performed with the “unfiltered” as well as the filtered dataset. The analysis of both datasets were very similar indicating that the removal the SNPs considered likely to be polymerase -induced artifacts was valid and did not influence the results of the neutrality tests. The confidence limits and p-values were obtained by coalescent simulations (10 000 replicates) without recombination. The number of unique recombination events and the cross-over positions were estimated according to the methods (RDP, GenConv, Chimera, MaxChi, Bootscan, Siscan, 3Seq, LARD) implemented in the software package RDP3 [37].

## Results

### Differences in genetic diversity and population structure between *H*. *contortus* and *T*. *circumcincta*

In order to examine and compare the population genetic structure of *H*. *contortus* and *T*. *circumcincta* on the same farms, seven UK farms were chosen on which there was a high prevalence of both species present (see Materials and Methods) (Fig. 1A).

Thirty individual L_1_ larvae were genotyped for *H*. *contortus* and *T*. *circumcincta* at each of ten microsatellite loci for each farm population. There were no major departures from linkage equilibrium for all pairwise combinations of loci in any population, indicating that alleles at these loci were randomly associating and not genetically linked. All farm populations were polymorphic at all loci for both species, with the number of alleles per locus ranging from 2 to 17 for *H*. *contortus* and from 3 to 19 for *T*. *circumcincta* (S4 and S5 Tables). Both parasites showed a high level of genetic diversity in all populations but *T*. *circumcincta* showed a higher level of overall diversity then *H*. *contortus*. The mean allele richness was 7.51 ±0.25 alleles per locus for *H*. *contortus* compared to 11.11 ±0.23 for *T*. *circumcincta* (Table 1). The expected heterozygosity (H_e_) was 0.670 for *H*. *contortus* (range: 0.631–0.694) compared to 0.823 for *T*. *circumcincta* (range: 0.797–0.840) with very little difference in overall genetic diversity between any of the seven farms (Table 1).

**Table 1:** Allelic diversity of microsatelllite loci and β-tubulin from *H*. *contortus* and *T*. *circumcincta* from each of the seven study farms and neutrality test statistics.

Diversity (Microsatellite data)			Diversity (β-tubulin sequence data)									Neutrality tests (β-tubulin sequence data)			
H. contortus															
	H_E_	A	n_Tot_	h_Sus_	h_Res_	h_Tot_	H_d_	S	k	π	θ_S_	D	p	H	p
Farm 37	0.6634	7.5	20	0	5	5	0.442	50	9.279	0.0101	0.0154	-1.53	0.046*	-16.95	0.012*
Farm 54	0.6308	6.9	20	0	2	2	0.521	2	1.042	0.0011	0.0006	1.99	0.985	-0.02	0.281
Farm 86	0.6807	8.5	20	2	3	5	0.511	42	11.400	0.0124	0.0132	-0.15	0.48	-7.85	0.054
Farm 95	0.6943	7.8	20	0	1	1	0.000	0	0	0	na				
Farm 101	0.6894	7.4	20	1	2	3	0.353	36	4.005	0.0044	0.0113	-2.41	0.000***	-11.96	0.000***
Farm 102	0.6498	6.5	20	8	0	8	0.742	53	20.842	0.0227	0.0163	1.20	0.917	-8.79	0.126
Farm 110	0.6810	8.0	20	5	1	6	0.747	55	19.679	0.0214	0.0169	0.82	0.841	-5.31	0.159
Resistant			104			5	0.506	50	3.319	0.0036	0.0104	-2.14	0.001***	-23.34	0.001***
Susceptible			36			10	0.743	55	22.273	0.0243	0.0145	2.06	0.990	-7.09	0.157
Total	0.6700	7.51	140			15	0.712	58	12.774	0.0139	0.0115	0.42	0.425	-14.60	0.031*
T. circumcincta															
	H_E_	A	n_Tot_	h_Sus_	h_Res_	h_Tot_	H_d_	S	k	π	θ_S_	D	P	H	p
Farm 37	0.8394	12.1	20	0	10	10	0.916	56	17.016	0.0190	0.0187	0.28	0.676	-19.38	0.025*
Farm 54	0.7966	10.9	20	6	5	11	0.9	85	24.168	0.0269	0.0275	-0.06	0.521	-22.73	0.039*
Farm 86	0.8326	10.7	20	1	4	5	0.505	52	12.984	0.0145	0.0172	-0.44	0.381	-19.67	0.018*
Farm 95	0.8395	11.1	20	0	4	4	0.284	14	1.758	0.0020	0.0043	-2.03	0.003***	-5.73	0.021*
Farm 101	0.8214	10.5	20	3	7	10	0.889	59	25.926	0.0289	0.0201	1.95	0.987	-5.94	0.192
Farm 102	0.8175	10.7	20	8	3	11	0.921	88	32.789	0.0366	0.0283	1.08	0.888	-5.52	0.202
Farm 110	0.8130	11.8	20	1	8	9	0.747	79	25.184	0.0281	0.0258	0.38	0.376	-15.18	0.090
Resistant			110			28	0.878	98	19.716	0.0220	0.0209	0.08	0.61	-28.07	0.016*
Susceptible			30			15	0.926	88	28.002	0.0312	0.0253	0.86	0.857	-14.87	0.100
Total	0.8229	11.11	140			43	0.918	107	23.651	0.0264	0.0216	0.50	0.788	-27.32	0.038*

H_E_, Expected heterozygosity; A, mean number of alleles per locus; h_Sus_, total number of susceptible haplotypes; h_Res_, total number of resistant haplotypes;

h_Tot_, total number of haplotypes; H_d_, Gene diversity; S, number of segregating sites; k, mean number of pairwise differences; π, nucleotide diversity;

θ_S_, Mutation parameter based on infinite site equilibrium model and the number of segregating sites; *D*, Tajima’s *D* test statistic; *H*, Fay and Wu’s *H* test statistic. Statistical significant departure from neutrality determined with the use of simulations of the coalescent at p<0.05 *, p<0.01 **, and p<0.005***.

There was a significant departure from Hardy-Weinberg equilibrium, even after Bonferroni correction, in addition to relatively high inbreeding coefficient values (F_IS_) for 43 out of the 70 loci/farm combinations for *H*. *contortus* and 52 out of the 70 loci/farm combinations for *T*. *circumcincta* (S4 and S5 Tables). The presence of null alleles for microsatellite loci has been previously reported for these two parasite species and is the likely reason for departures from Hardy-Weinberg Equilibrium [14,20,38, 39]. This was confirmed for two loci (Hc22c03 and Hcms40) where sequencing flanking sequence revealed deleted sequence encompassing primer sites in the ‘suspected’ null alleles.

AMOVA analysis estimated that the percentage of variation that partitioned between farm populations was 10-fold less for *T*. *circumcincta* (0.24%) compared to *H*. *contortus* (2.84%) suggesting greater geographical sub-structuring of *H*. *contortus* than *T*. *circumcincta*. This was reflected by the pairwise F_ST_ estimates calculated between each of the seven populations (Fig. 1B). Only two out of the possible 21 pairwise comparisons between farms showed statistically significant but low differentiation for *T*. *circumcincta* (Fig. 1B). These were between farms 101 (E. Sussex) and 110 (Kent) (F_ST_ = 0.0269) and between farms 101 (E. Sussex) and 54 (Devon) (F_ST_ = 0.0340). In contrast, 10 out of 21 possible pairwise comparisons showed significant differentiation for *H*. *contortus* (Fig. 1B) with F_ST_ values ranging from 0.0198 (between farms 37 (Gloucester) and 86 (Middlesex)) to 0.0757 (between farms 54 (Devon) and 110 (Kent)). However, no single farm population was significantly genetically differentiated from all of the other six populations. Estimates of F_ST_ after null allele correction were similar to uncorrected estimates (Fig. 1B). The SplitsTree4 network confirms the lack of genetic differentiation between populations for *T*. *circumcincta* but suggests moderate genetic differentiation between some of the *H*. *contortus* populations (Fig. 1C).

### Differences in benzimidazole resistance associated mutations between *H*. *contortus* and *T*. *circumcincta*

**Fig 2:**
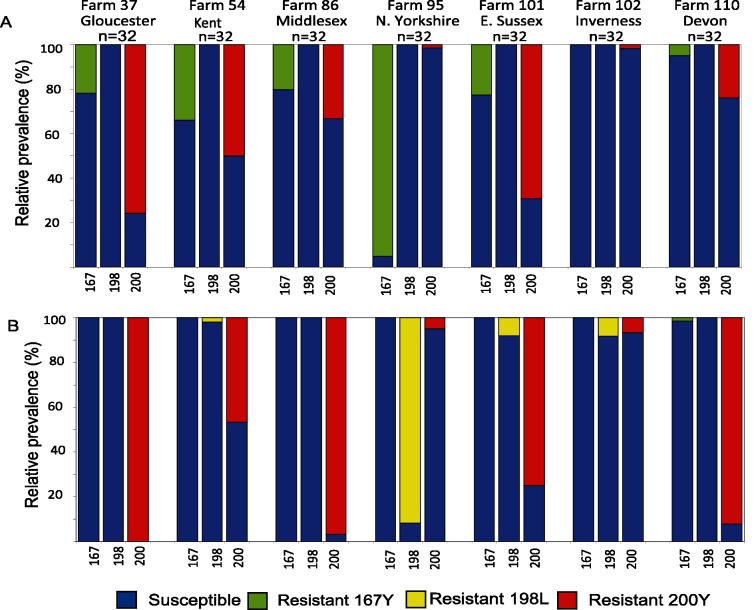
The relative proportions of isotype-1 β-tubulin alleles encoding resistance-conferring polymorphisms at F167Y, E198L or F200Y. Populations for (A) *H*. *contortus* and (B) *T*. *circumcincta*, consisting of 30 or 32 individual worms, were pyrosequence genotyped at the three benzimidzole resistance associated SNPs in separate assays to determine the proportion of each SNP in each population.

The same *H*. *contortus* and *T*. *circumcincta* larvae that were genotyped for the microsatellite loci were individually genotyped for the three currently known benzimidazole resistance associated polymorphisms in the isotype-1 β-tubulin gene (F167Y, E198A and F200Y) using pyrosequence genotyping. The F167Y (T**T**C>T**A**C) and F200Y (T**T**C>T**A**C) resistance polymorphisms were detected in both species and, although the substitution of glutamic acid (E) for alanine (A) at position 198 (G**A**A>G**C**A) was not identified in either species, a change in codon 198 from GAA (or GAG) to TTA resulting in the substitution of glutamic acid (E) for leucine (L) was identified in *T*. *circumcincta*. (Fig. 2).

In the case of *H*. *contortus*, the F200Y resistance polymorphism was present on all seven farms and, with the exception of farm 95 (N. Yorkshire), it was the most common resistance polymorphism in *H*. *contortus* (36.3% overall). The P167^Y^ polymorphism was also common, being present in six out of seven farms, in some cases at relatively high frequency (Fig. 2). Farm 102 (Inverness) was the only farm on which the majority of *H*. *contortus* had susceptible genotypes at all three resistance associated positions.

In the case of *T*. *circumcincta*, the F200Y resistance polymorphism was also extremely common, with a prevalence of >46% in five out of the seven farms. It was at fixation on farm 37 (Gloucester) and close to fixation on two other farms, farm 86 (Middlesex) and farm 110 (Kent). However, unlike the situation in *H*. *contortus*, the F167Y resistance polymorphism was found at very low frequency (1.7% overall) on just one farm, 110 (Kent) (Fig. 2). In contrast, the E198L polymorphism was present on four out of the seven farms. Although it was at relatively low frequency in three of these (2.0–8.8%), it was very high frequency on farm 95 (N. Yorkshire), being close to fixation (91.7%). As with *H*. *contortus*, the only farm on which the majority of *T*. *circumcincta* (93.3%) had susceptible variants at all three positions was farm 102 (Inverness) (Fig. 2). No individual worms of either species were homozygous for more than one resistance polymorphism.

### Isotype-1 β-tubulin locus diversity and signatures of selection

**Fig 3:**
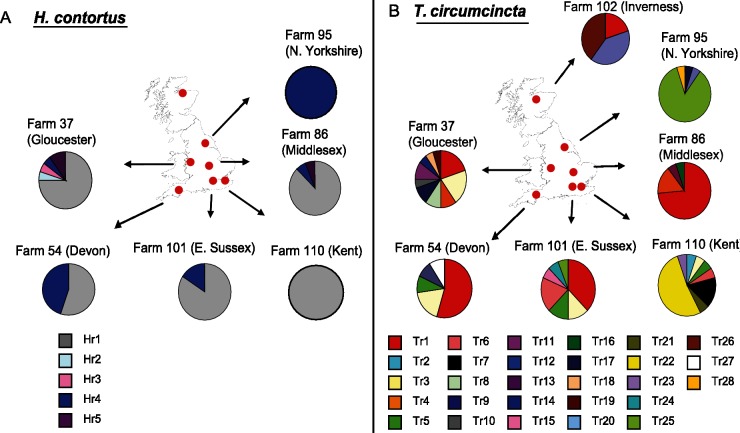
Relative frequency of individual resistant haplotypes in seven UK sheep parasite populations. A total number of five resistant haplotypes for (A) *H*. *contortus* and twenty eight resistant haplotypes for (B) *T*. *circumcincta* were identified. Susceptible haplotypes are not included this figure but are show in S3 Fig.

In order to sample the diversity of the isotype-1 β-tubulin locus and look for evidence of selection, twenty cloned copies of the region encompassing the P167, P198 and P200 positions (922bp sequence for *H*. *contortus* and 940bp sequence for *T*. *circumcincta*), were sequenced for each parasite species from each of the seven farms (a total of 140 sequences for each species). Polymorphisms likely to be due to PCR induced mutations were removed from the dataset to give a conservative estimate of allelic diversity (see Materials and Methods). The isotype-1 β-tubulin genetic diversity was high in both species but was greater in *T*. *circumcincta* with higher numbers of polymorphic sites, total number of haplotypes, pairwise differences and higher values for nucleotide and gene diversity. The distribution of the resistant haplotypes is shown in Fig. 3 and the full data for both resistant and susceptible haplotypes is shown in (Table 1 and S3 Fig.). The GenBank accession numbers for the sequences are given in S6 Table. There was a total of fifteen different isotype-1 β-tubulin haplotypes for *H*. *contortus* and forty three different haplotypes for *T*. *circumcincta* across the seven farms. Five out of the fifteen *H*. *contortus* isotype-1 β-tubulin haplotypes (33.3%) encoded either a P167Y or P200Y resistance polymorphism (resistance alleles). Twenty eight out of forty three *T*. *circumcincta* haplotypes (65.1%) encoded either a P198L or P200Y resistance polymorphism (resistance alleles) (Table 1 and S3 Fig.).

There was clear evidence of selection at the isotype-1 β-tubulin locus in both species when resistance and susceptible haplotypes were compared for the full dataset overall. Pairwise F_ST_ values estimated from pyrosequence genotypes at the benzimidazole resistance-associated SNPs revealed considerable genetic differentiation between the populations: 18 of 21 and 13 of 21 pairwise comparisons were statistically significant for *H*.*contortus* and *T*. *circumcincta* respectively (S4 Fig.). This was compared to the extremely low levels of genetic differentiation identified using the neutral loci (Fig. 1B). In addition, for *H*. *contortus*, there was an overall reduction in gene diversity for resistant (0.506) relative to susceptible (0.743) isotype-1 β-tubulin haplotypes (Table 1). Moreover, there was a statistically significant departure from neutrality for both Tajima’s *D* (-2.14, p-value<0.005) and Fay and Wu’s *H* statistic (-23.34, p<0.001) for *H*. *contortus* resistant haplotypes but not for susceptible haplotypes (2.06, p>0.05 and -7.09, p>0.05 respectively) suggesting selection at this locus (Table 1). The statistically significant positive estimate of Tajima’s *D* (2.06, p<0.01) for the susceptible haplotypes is consistent with the moderate degree of sub-structure identified by the neutral microsatellite marker analysis (Fig. 1). For *T*. *circumcincta*, some evidence of selection was still apparent in spite of the high level of haplotype diversity. Although gene diversity estimates of *T*. *circumcincta* resistant and susceptible haplotypes were similar (0.878, Resistant; 0.926, Susceptible) and Tajimas’s *D* was not statistically significant, the estimates of Fay and Wu’s *H* statistic showed statistically significant departure from neutrality for resistant haplotypes (-28.069, p<0.05) but not for susceptible haplotypes (-14.87, p>0.05) (Table 1).

Genetic diversity was also assessed for the two parasite species at the individual farm level (Table 1, Fig. 3, S3 Fig.). There was a significant departure of the *H* statistic from neutrality for farms 37 (Gloucester) and farm 101 (E. Sussex). Farm 86 (Middlesex) was close to significance (p = 0.054) and a single haplotype was at fixation on farm 95 (N. Yorkshire). In contrast, higher gene diversity was observed on farms where there was a low frequency of resistance mutations (farms 102 (Inverness) and 110 (Kent)). Hence, for the four out of the five farms which had a high frequency of resistance mutations, there was clear evidence of selection at the isotype-1 β-tubulin for *H*. *contortus*.

In the case of *T*. *circumcincta*, in spite of the extremely high overall level of sequence diversity, evidence of selection at the isotype-1 β-tubulin locus was also detectable on some of the farms with a high frequency of resistance mutations. For farms 86 (Middlesex) and 95 (N. Yorkshire), a significant *H* statistic and lower gene diversity estimates clearly indicate selection (Table 1). In the case of farm 37 (Gloucester), even though 10 different resistance haplotypes were present, producing a high estimate of gene diversity (0.916), a signature of selection was still detectable by the *H* statistic (19.38, p = 0.025). The only farm of the seven that had a predominantly susceptible *T*. *circumcincta* population, farm 102 (Inverness), had the highest level of haplotype diversity and the least evidence of selection on the neutrality tests (Table 1).

### Phylogenetic relationships between isotype-1 β-tubulin haplotypes

**Fig 4:**
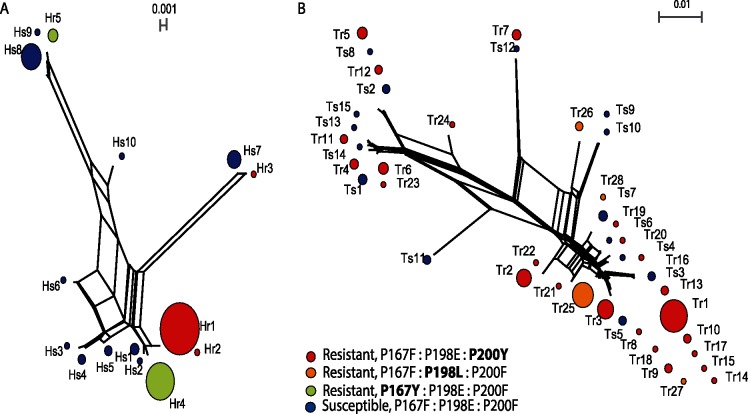
Network analysis of sequences of isotype-1 β-tubulin sequences. Split networks were generated with the neighbour-net method of SplitsTrees4 from (A) *H*. *contortus* sequences and (B) *T*. *circumcincta* sequences. The circles in Split networks represent the different haplotype and the size of the circles is proportional to the frequency in the population. The colours define the resistant haplotypes across positions P167, P198 and P200 (P167F:P198E:P200F, Susceptible = blue; P167F:P198E:P200Y, Resistant at P200 = Red; P167Y:P198E:P200F, Resistant at P167 = green; P167F:P198L:P200F, Resistant at P198 = orange).

SplitsTree4 networks were produced to examine the phylogenetic relationship between isotype-1 β -tubulin haplotypes (Fig. 4). For both species, the resistance haplotypes are polyphyletic, being distributed across the entire network consistent with a hypothesis that resistance mutations have appeared on different ancestral susceptible haplotypes (Fig. 4).

For *H*. *contortus*, resistance haplotypes are present in three separate branches of the SplitsTree4 network (Fig. 4A); group 1 (Hr1/Hr2/Hr4), group 2 (Hr3) and Group 3 (Hr5). Group 1 (Hr1/Hr2/Hr4) includes haplotypes with either the P200Y or P167Y mutations. While the network shows the frequency of the resistance and susceptible haplotypes across all farms in the study (Fig. 4A), Fig. 3 shows the frequency of resistance haplotypes on each farm. The resistance haplotypes with the highest frequency for *H*. *contortus* are Hr1 (P200Y) and Hr4 (P167Y) and both of these are identical to the susceptible haplotype Hs1 except for the resistant-associated SNPs. Hr1 was the most frequent haplotype on five out of seven farms (farm 37 (Gloucester), farm 54 (Devon), farm 86 (Middlesex), farm 101 (E. Sussex) and farm 110 (Kent)) and Hr4 was identified on five farms (farm 95 (N. Yorkshire), farm 37 (Gloucester), farm 54 (Devon), Farm 86 (Middlesex) and farm 101 (E. Sussex)) (Fig. 3). In contrast, the putative ancestral susceptible haplotype Hs1 was present on just 2 farms (farms 102 (Inverness) and 110 (Kent)) (Fig. 3). Farm 37 (Gloucester) has the most diversity of resistance haplotype for *H*. *contortus* with all five resistant haplotypes being present (Fig. 3). Conversely, farm 95 (N. Yorkshire) has the least diversity of resistance haplotypes with Hr4 being almost at fixation. There are just two farms that possess a high proportion of *H*. *contortus* susceptible haplotypes: farm 110 (Kent, 0.67) and farm 102 (Inverness, 0.98). The susceptible haplotypes on both these farms appear highly diverse (S3 Fig.) and distantly-related to each other (Fig. 4A).

For *T*. *circumcincta*, the extremely high level of haplotype diversity leads to a more complicated set of phylogenetic relationships (Fig. 4B). Nevertheless, the overall pattern is similar to that described for *H*. *contortus* with resistance haplotypes occurring all across the SplitsTree4 network (Fig. 4B). This again suggests independent origins of multiple resistance mutations. The most prevalent resistance haplotype for *T*. *circumcincta*, Tr1 was represented on five of the seven farms but not always at the highest frequency (Fig. 3). The exceptions being farm 110 (Kent) and farm 95 (N. Yorkshire), where the Tr1 haplotype was not identified despite a high level of resistance haplotypes (95% and 100% respectively). The resistance haplotype, Tr25 (carrying the P198L polymorphism) was found on two farms with 94% of all Tr25 haplotypes being identified on farm 95 (N. Yorkshire). The two farms on which there are relatively high frequencies of susceptible haplotypes for *T*. *circumcincta*, farm 54 (Devon, frequency 0.5) and farm 102 (Inverness, frequency 0.93), the susceptible haplotypes are highly diverse (S3 Fig.) and distantly-related to each other (Fig. 4B).

Many other methods for network estimation including neighbor-net (SplitsTree4) networks have been reviewed by Posada and Crandall [40] and evaluated in Woolley *et al*., 2008 [41] who states “when the phylogenetic relationship of any set of sequences is being inferred, it is important that several methods be used and their inferences inspected and compared for discrepancies.” Consequently in addition to a method based on genetic distance (Neighbor-net, as implemented in SplitsTree4) two other methods were also chosen for this study: one character-based (Median-Joining in Network 4.6.1, see S5 Fig.) and one based on Statistical Parsimony (TCS, see S6 Fig.). Similar results were seen in these networks (Median-Joining and Statistical Parsimony) as was seen with the Neighbor-net networks for both species.

### Evidence of genetic recombination between different isotype-1 β-tubulin haplotypes

We investigated the extent to which genetic recombination has played a role in generating the haplotypic diversity. For *H*. *contortus*, just one haplotype (Hs10) of the total number of isotype-1 β-tubulin haplotypes (15) was identified as a recombinant (S7A Fig.). Although recombination was detected, it does not account for the majority of the haplotypic diversity for *H*. *contortus*. In contrast, for *T*. *circumcincta* 22 of the total number of isotype-1 β-tubulin haplotypes (43) were identified as possible recombinants resulting from just 5 unique recombination events (S7B Fig.). It is notable that three of the four hapotypes carrying the P185L polymorphism are clearly related by recombination on the basis of this analysis. Both re-current mutation and recombination contribute to the haplotypic diversity present in *T*. *circumcincta*.

## Discussion

For many years the widespread development of anthelmintic resistance in livestock parasites was assumed to be due to selection acting on ancestral mutations that pre-dated the onset of drug use [8,42]. This hypothesis was consistent with the prevailing view that rapid adaptive change in eukaryotes was limited by the availability of new mutations [12]. More recently this “pre-existing mutation” hypothesis for anthelmintic resistance has been challenged in several papers that have suggested that recurrent mutations occurring after the onset of selection may also be important [9,39, 43]. In the discussion below, we explain how the results presented here are consistent with this latter hypothesis. We propose a model in which multiple independent anthelmintic resistance mutations recurrently arise and, when combined with migration due to animal movement, produce a complex pattern of hard and soft selective sweeps. This model explains why anthelmintic resistance has become so widespread in the trichostrongylid nematodes and provides important information for genome-wide studies aimed at identifying anthelmintic resistance mutations in these and other parasitic nematodes.

### Differences in genetic diversity and population structure of *H*. *contortus* and *T*. *circumcincta* reflect epidemiological differences on UK farms

The first major study of the population genetics of *H*. *contortus* and *T*. *circumcincta* used mitochondrial DNA analysis of samples obtained from several locations in North America [44]. This study detected no population sub-structuring between locations and concluded that high levels of gene flow and a larger overall effective population size (N_e_) were responsible. However, more recent microsatellite marker studies have suggested that the situation is more complex. Although *H*. *contortus* and *T*. *circumcincta* are phylogenetically close, and their genetics similar, they appear to differ in population structure [14, 20]. Whilst a lack of genetic sub-structuring has been confirmed for *T*. *circumcincta* in both the UK and France [20, 39, 45], some genetic sub-structuring of *H*. *contortus* has been reported in France and Sweden [39,46]. Most notably, a microsatellite marker study on goat farms in France detected between-farm population divergence for *H*. *contortus* but not for *T*. *circumcincta* [39]. In the French study, farms were specifically chosen that had been closed to animal movement for at least 15 years in order to remove the effects of parasite migration. In the study reported here, we have sampled UK farms that are largely open to animal movement, a situation that is more typical of ruminant livestock systems worldwide. In addition, we have sampled the two parasite species from the same individual animals on each of seven farms in order to compare the two species without any confounding environmental differences such as climate, farm management or individual host effects. We have found that, even in the presence of animal movement, *H*. *contortus* has significantly lower genetic diversity and more population sub-structuring than *T*. *circumcincta*, both at neutral microsatellite markers and at the isotype-1 β-tubulin locus. Given our study design, we propose these genetic differences must be related to differences in parasite life-history traits.

The evolutionary origins of *H*. *contortus* are in sub-Saharan Africa from where it has been transported around the globe by livestock movement over the last several hundred years [47,48]. Consequently, its larval stages are poorly adapted for winter pasture survival in temperate regions and so persistence during the colder months is largely confined to the parasitic stages inside the host [49]. This is likely to lead to population bottlenecks, particularly in the UK, where anthelmintic drug treatments are commonly given to all animals, including in the early spring when few parasites are present on the pasture. In contrast, *T*. *circumcincta* can potentially maintain a larger population size since it is indigenous to temperate regions and so its free-living stages are better adapted for winter pasture survival. This difference in the evolutionary history of the two parasites is clearly reflected in their epidemiological patterns in the UK. *H*. *contortus* is generally only detected at low levels on UK farms but its high fecundity can result in rapid expansion of numbers causing acute clinical disease outbreaks during the summer [13,47]. In contrast, *T*. *circumcincta* is consistently present in high numbers from year to year on all UK farms and this is typical of other temperate regions [13,49–51].

### Differences in the benzimidazole resistance mutations present in *H*. *contortus* and *T*. *circumcincta* on UK farms

Three different single nucleotide polymorphisms (SNP) in the isotype-1 β-tubulin gene have been previously associated with benzimidazole resistance in these parasite species. TTC to TAC transversions at codons 200 and 167 resulting in substitution of phenylalanine with tyrosine (F200Y and F167Y) has been reported in both *H*. *contortus* [6,7,52, 53] and *T*. *circumcincta* [2,3,7–9,39]. In addition, a GAA to GCA transversion at codon 198 leading to a substitution of glutamic acid with alanine (E198A) has been reported in *H*. *contortus* [4,27]. The F200Y and F167Y substitutions were identified in this study for both *H*. *contortus* and *T*. *circumcincta*. The finding that the F200Y is the most common substitution in both species (36.32% and 60.31% overall frequencies respectively) is consistent with a variety of previous studies across the world [28,38,39, 52, 54]. However, the observation that F167Y was also very frequent in *H*. *contortus* (28.8% overall and almost at fixation on farm 95, N. Yorkshire) differs from previous reports where the F167Y has generally been only found at low frequency [39,52].

The identification of a substitution of glutamic acid (GAA or GAG) with leucine (TTA) at position 198 in *T*. *circumcincta* on four out of the seven farms (15.8% overall) was of particular interest. To our knowledge this particular substitution has been identified only once previously, in an isolate from New Zealand [55]. This polymorphism was found at very high frequency on a single haplotype on farm 95 (N. Yorkshire) with strong evidence of selection (based on neutrality tests) consistent with a role in benzimidazole resistance.

### Benzimidazole resistance in *H*. *contortus* and *T*. *circumcincta* on UK farms involves both hard and soft selective sweeps

Understanding the nature of adaptive changes occurring in response to anthelmintic selection in parasite populations is key to understanding the origin and spread of resistance mutations. Rapid adaptation in response to selection results in so-called “selective sweeps” at the loci under selection. There are essentially two different types of selective sweep [12,56,54]. The classic “hard selective” sweep is characterised by a single resistance haplotype in the populations(s) resulting from a single mutation arising and sweeping through the population(s) eventually reaching fixation. Soft selective sweeps are characterised by the presence of multiple resistance haplotypes in the population(s) and can arise in two ways. First, from recurrent *de novo* mutations appearing on different susceptible haplotype backgrounds after the onset of the selection. Second, from polymorphisms already present in the standing genetic variation before the onset of selection. It has recently, been suggested that soft selective sweeps may be more common in eukaryotes than previously recognized, particularly for organisms with large census population sizes [12]. For example, resistance to organophosphate insecticides in *Drosophila melanogaster* and *Lucilia cuprina* involve multiple independent resistance alleles at the acetylcholinesterase and esterase loci respectively [57,58,56]. In reality, the difference between hard and soft selective sweeps is not absolute with selective sweeps differing in number and relative frequency of the different resistance haplotypes in a population [12].

The high diversity of resistance haplotypes described in this study is truly remarkable when compared to studies of drug resistance in other eukaryotic systems. Five and twenty eight resistance haplotypes were found for *H*. *contortus* and *T*. *circumcincta* respectively on just seven farms. This is much higher than previously reported even for insecticide resistance in insects such as *D*. *melanogaster* or *Anopheles* mosquitos [57,59–61]. For *H*. *contortus*, although both hard and soft selective sweeps are present there is a predominance of hard sweeps (Fig. 3A and S3 Fig.). On farms 95 (N. Yorkshire) and 110 (Kent) a single resistant haplotype was detected characteristic of a classical “hard” selective sweep (Fig. 3A and S3 Fig.). On farms 37 (Gloucester), 86 (Middlesex) and 101 (E. Sussex), although multiple resistance haplotypes are present, one of these predominates at high frequency in each case. However, in contrast, on farm 54 (Devon) there are two discrete resistance haplotypes at almost equal frequency characteristic of a “soft” selective sweep. For *T*. *circumcincta*, the selective sweeps are generally much softer than for *H*. *contortus*. The most extreme example is farm 37 (Gloucester) on which there are a remarkable ten different resistance haplotypes present at similar frequency. This is consistent with the overall higher level of genetic diversity of *T*. *circumcincta* providing a greater supply of resistance mutations. Even so, a predominantly hard selective sweep is present on farm 95 (N. Yorkshire) a single haplotype predominates at high frequency.

### The case for the importance of recurrent mutation in the emergence of benzimidazole drug resistance

In soft selective sweeps, it can be difficult to distinguish between adaptive mutations derived from the standing genetic variation versus those derived from recurrent *de novo* mutation occurring after the onset selection [12]. “Ancient” polymorphisms are present on many haplotype backgrounds due to historical recombination and so, if selected, would lead to a high level of haplotypic diversity and a soft selective sweep. Hence, the presence of a soft selective sweep doesn’t, in itself, prove that mutations are recurrently appearing in parasite populations subsequent to the onset of selection. However, the striking differences we see in the selective sweeps between farms is consistent with the hypothesis that recurrent mutations occur. This is most obvious for *T*. *circumcincta*; since there is little genetic sub-structuring of this parasite between farms (based on neutral microsatellite markers), the standing genetic variation should be similar on each farm. Consequently, if the standing genetic variation were the only source of resistance mutations, one would predict that broadly similar selective sweeps and resistance haplotypes would be present on each farm. However, this is clearly not the case (Fig. 3B and S3 Fig.). For example, *T*. *circumcincta* populations on farms 37 and 95 have little genetic differentiation based on neutral markers (F_ST_; -0.0023) but have dramatically different selective sweeps; ten different resistance haplotypes for farm 37 and a four different resistance haplotypes on farm 95 without a single haplotype being shared. Likewise for *H*. *contortus*, for farms 37 and 95 there are completely different resistance haplotypes present in spite of very little genetic differentiation for neutral markers (F_ST_ 0.0087). The phylogenetic relationship of the resistance haplotypes is also consistent with a model of multiple independent origins of benzimidazole resistance mutations across the UK. In the case of *H*. *contortus*, there are at least 4 independent origins of the resistance haplotypes present on seven UK farms and an even larger number for *T*. *circumcincta*. In both cases, with the exception of the P198L substitution, our analysis detects few recent recombination events consistent with mutations appearing on divergent susceptible haplotypes.

Given the large population sizes of trichostrongylid nematodes, we should not be surprised that independent resistance mutations can repeatedly arise and become selected in these organisms. The population parameter = 2N_e_ describes the rate at which new mutations arise in a population where N_e_ = effective population size and = the per site mutations rate [62]. It has been suggested that if is greater than 0.1, then soft selective sweeps are likely to occur [12,56]. A population size of 10^7^ would be predicted to be sufficient for recurrent mutations to repeatedly arise and produce soft selective sweeps if the mutation rate is similar to that of *C*. *elegans* (10^–8^ mutations per base per generation). We, and others, have previously pointed out that a single flock of a few hundred sheep can potentially contaminate pastures with billions of eggs every week [9,43, 47]. Although, the majority of these larvae on the pasture never make it into the host, it is very likely that N_e_ for these organisms is more than adequate for soft selective sweeps to occur even at the individual farm level.

### The role of migration in determining the spread of resistance mutations and the complex pattern of the selective sweeps

There is a large amount of movement of sheep between farms in the UK and so it is likely that migration of resistance haplotypes plays an important role in the spread of resistance. However, the presence of the same resistance haplotype on two or more farms cannot, in itself, be taken as conclusive evidence of spread. It is quite possible that a resistance mutation could independently appear on the same susceptible haplotype background at different locations particularly given the low level of population sub-structuring of parasite populations between farms. However there are a number of pieces of evidence to suggest that the migration of anthelmintic resistance mutations between farms is an important factor in the spread of anthelmintic resistance in the UK.

Firstly, although there are major differences in the selective sweeps and haplotypes found on the seven different UK farms (as described above), it is notable that the same *H*. *contortus* resistance haplotype (Hr1) predominates on five of the seven farms. This contrasts with the results of a previous study of ten goat farms in France that were closed to animal movement for 15 to 30 years [7,8,39]. In that case, a different *H*. *contortus* resistant haplotype generally predominated on each farm. Hence, it seems much more likely that animal movement has contributed to the spread of the Hr1 haplotype on UK farms rather than it appearing independently so many times. Secondly, in the French study, only one, or at most two, resistance haplotypes were found on each farm whereas there was a much larger number of haplotypes present on most of the UK farms in this study. Although, the number of sequences examined per farm varied for the French study, thirty *T*. *circumcincta* alleles were sequenced on two of the closed goat farms and yet only a single resistance haplotype was detected in each case (a different haplotype was present on each farm) [39]. This may suggest that the high diversity of haplotypes seen on UK farms is at least partly due to the migration of resistance haplotypes due to animal movement. Thirdly, it seems unlikely that the E198L substitution has appeared independently on four out of the seven farms in this study given that it has never been previously reported and that two different SNP substitutions of the susceptible codon (P198E; GAA or GAG) are required to produce the putative resistance codon (P198L; TTA). It seems more likely that this P198L substitution has appeared once and spread between farms by migration. This hypothesis is supported by the observation that three of the four P198L haplotypes are related by recombination (S7B Fig.).

### A signature of selection is detectable at the isotype-1 β-tubulin locus in spite of high allelic diversity

The application of genome-wide approaches to identify regions of the genome under selection is increasingly feasible for parasitic nematodes due to the recent improvements of sequencing technologies and reference genomes. However, such approaches need a good understanding of the signature of selection. Hard selective sweeps are the simplest to detect as they are characterised by a dramatic loss of polymorphism around the locus under selection due to the “hitchhiking effect” [11,63]. Hence, simple measures of genetic diversity and neutrality such as H_d_, Tajima’s *D* and Fay and Wu’s *H* should reliably detect hard selective sweeps. Soft selective sweeps are more difficult to detect since loss of polymorphism around the selected locus is often subtle or even absent although linkage disequilibrium can be strong [43,42,56,64]. However, our results suggest that, in spite of the overall complexity of the selective sweeps associated with benzimidazole resistance on UK farms, evidence of selection can still be detected in many cases. For *H*. *contortus*, where the selective sweeps on most of the individual farms was effectively hard, selection could be detected by significant departures from neutrality by Fay and Wu’s *H* statistic on 4 out of the 5 farms which had a high frequency of resistance mutations [farms 37 (Gloucester), 86 (Middlesex), 95 (N. Yorkshire) and 101 (E. Sussex)]. For farm 54 (Devon) where two resistance haplotypes are present at almost equal frequency, although *D* and *H* statistics do not depart from neutrality, selection is still reflected by a loss in overall diversity. Even for *T*. *circumcincta*, where the selective sweep is generally much softer, selection could be detected on two of the farms, 95 (N. Yorkshire) and 86 (Devon). As a final point, it is important to acknowledge that, negative values for Tajima’s *D* can sometimes result from population expansion following an historical population bottleneck. However, there are a number of reasons why this is unlikely to have occurred in this case. First, the use of the *H* statistic helps distinguish between the effects of population dynamics and selection since it is less sensitive to the effects of population expansion [36]. Second, and more importantly, it is difficult to explain the dramatic allele frequency differences, and departures from neutrality, at the isotype-1 β-tubulin locus when all 10 microsatellites show little or no between farm differences in allele frequency (since all loci will have experienced the same demographic effects). Thirdly, the population biology of these organisms makes it unlikely that severe population bottlenecks have occurred. Although some bottlenecking could occur with *Haemonchus contortus* this would not be expected to be severe. This is particularly the case for *T*. *circumcincta* in the UK where large numbers of larvae survive on the pasture year round and so many genotypes would persist at the farm level even if drug treatments in the host were highly effective. Hence, there is a strong balance of evidence for selection at the isotype-1 β-tubulin locus in both parasite species.

### Conclusion

The seven farms we have examined are typical of those in many parts of the world in terms of animal movement, husbandry and parasite control practices. In this system, our data supports a model of parallel adaptation in response to selection at separate locations with subsequent mixing of independently derived resistance mutations due to parasite migration between farms. In the case of *H*. *contortus*, we identified resistance mutations from at least four independent origins and almost certainly more for *T*. *circumcincta*. The most common haplotypes were identified on several of the seven farms selected from different regions of the UK and so these haplotypes are likely to be amongst the most common present in the UK parasite community. Nevertheless, it seems likely that additional independently derived haplotypes would be identified if more farms were sampled. Our model, in which recurrent resistance mutations commonly arise, suggests that for this group of parasites anthelmintic resistance will be a likely consequence of intensive drug use. This explains the increasingly widespread resistance in strongylid nematodes of domestic animals and, although there are important differences for the potential spread of resistance in human populations, has implications for the potential consequences of intensive drug use for human parasite control.

## Supporting Information

S1 Fig Relative prevalence of *H*. *contortus* and *T*. *circumcincta* on a sub-set of 20 farms. Prevalence estimation based on the amplification of 90 L_1_ larvae per farm with the rDNA ITS-2 species-specific PCR assays.
(EPS)

S2 Fig The distribution of single nucleotide polymorphism across the PCR amplified isotype-1 β-tubulin gene among 140 cloned *H*. *contortus* sequences (A) and 140 cloned *T*. *circumcincta* sequences (B). The position of the single nucleotide polymorphisms along the 922bp sequence for *H*. *contortus* and the 940bp sequence for *T*. *circumcincta*, is shown along the X-axis and the number of sequences that contain the SNP is plotted on the Y-axis. Grey shaded areas indicate exons.
(EPS)

S3 Fig Frequency distributions of isotype-1 β-tubulin alleles identified for *H*. *contortus* and *T*. *circumcinta*. The individual resistant and susceptible alleles are listed on the X-axis and the y-axis represents the number of sequences corresponding to each allele identified from the seven farms.
(EPS)

S4 Fig Pairwise F_ST_ values based on pyrosequence genotyping of the benzimidazole resistance-associated SNPs in the isotype-1 β-tubulin locus for *H*. *contortus* and *T*. *circumcincta*. Values underlined and in bold show significant differences (p < 0.002).
(EPS)

S5 Fig Median Joining network generated in Network 4.6.1 (Fluxus Technology Ltd.) from multi-aligned sequences for *H*. *contortus* (A) and *T*. *circumcincta* (B). A full median network containing all possible shortest trees was generated by setting the epsilon parameter equal to the greatest weighted distance (epsilon = 10). All unnecessary median vectors and links were removed with the MP option [32]. The size of each sequence node is proportional to frequency of the haplotype in the dataset and the colors indicate the farm(s) on which the haplotype was present. Small black dots represent median vectors. Number of mutations separating adjacent sequence nodes and or median vectors is indicated along connecting branches. The most probable ancestral node was determined by rooting the network to a closely related outgroup. A *T*. *circumcincta* sequence was used to root the *H*. *contortus* network and vice versa. The text of the haplotype names is coloured to reflect the resistance genotype at P167, P198 and P200 positions (P167F:P198E:P200F, Susceptible = blue; P167F:P198E:P200Y, Resistant at P200 = Red; P167Y:P198E:P200F, Resistant at P167 = green; P167F:P198L:P200F, Resistant at P198 = orange).
(EPS)

S6 Fig TCS Network analysis of isotype-1 β-tubulin sequences for *H*. *contortus* and *T*. *circumcincta*. Networks were generated by the method of statistical parsimony implemented in TCS from (A) *H*. *contortus* sequences and (B) *T*. *circumcincta* sequences. Each branch represents a single nucleotide mutation and empty nodes are inferred haplotypes. A considerable degree of genetic differentiation exists between alleles and thus has resulted in several independent sub-networks for each species. The proposed “ancestral” haplotype for each sub-network has been marked with an asterisk. The size of the nodes is proportional to the allele frequency in the dataset and the colors indicate the farm(s) on which the haplotype was present as indicated in the key. The color of the text giving the haplotype names reflects the resistance mutation genotype at positions P167, P198 and P200 (P167F:P198E:P200F, Susceptible = blue text; P167F:P198E:P200Y, Resistant at P200 = Red text; P167Y:P198E:P200F, Resistant at P167 = green text; P167F:P198L:P200F, Resistant at P198 = orange text). The software handles gaps in a sequence as missing data and this has resulted in a total of 40 alleles rather than 43: Tr22 collapses to Tr2, Tr16 collapses to Tr1 and Ts14 collapses to Ts2.
(EPS)

S7 Fig Summary of those haplotypes predicted to be related by recombination on the basis of analysis performed by RDP3 suite of programs for (A) *H*. *contortus* and (B) *T*. *circumcincta* isotype-1 β-tubulin alleles. (EPS)

S1 Table Primers and thermocycling parameters for rDNA ITS-2 species specific PCR assays. (DOC)

S2 Table Summary of bioinformatic screen for microsatellites of *H*. *contortus* and *T*. *circumcincta* and their experimental screening against a range of genetic divergent isolates. (DOC)

S3 Table New microsatellites developed for population genetics analysis of *T*. *circumcincta* (Tc) and *H*. *contortus* (Hc). (DOC)

S4 Table Population genetic data for the seven UK populations of *H*. *contortus* based on panel of 10 microsatellite loci. (DOCX)

S5 Table Population genetic data for the seven UK populations of *T*. *circumcincta* based on 10 microsatellite loci. (DOCX)

S6 Table Nomenclature of isotype-1 β-tubulin alleles identified in this study with corresponding GenBank numbers. (DOCX)
