# Supplementary material for: The Emergence of Resistance to the Benzimidazole Anthlemintics in Parasitic Nematodes of Livestock Is Characterised by Multiple Independent Hard and Soft Selective Sweeps
Source: PLoS Negl Trop Dis. 2015 Feb 6;9(2):e0003494. doi: 10.1371/journal.pntd.0003494 (PMC4319741; doi:10.1371/journal.pntd.0003494)
Supplement: S1 Table — (DOC) [file pntd.0003494.s008.doc]

Supplementary Table S1 Primers and thermocycling parameters for rDNA ITS-2 species specific PCR assays

| Species | Amplicon size | T_A_ (˚C) | No. of cycles | Origin | Primers (5’→3’) |
| --- | --- | --- | --- | --- | --- |
| *T. circumcincta* | 421bp | 52 | 35 | Burgess *et al*., 2012 | TcF:ATACCGCATGGTGTGTACGG |
|  |  |  |  |  | TcR:CAGGAACGTTACGACGGTAAT |
| *T. colubriformis* | 165bp | 52 | 35 | Burgess *et al*., 2012 | TcolF: CCCGTTAGAGCTCTGTATA |
|  |  |  |  |  | TcolR: TGCGTACTCAACCACCACTAT |
| *C. curticei* | 143bp | 52 | 35 | Burgess *et al*., 2012 | CcF: TATACTACAGTGTGGCTAGCG |
|  |  |  |  |  | CcR: TCATACCATTCAGAAATGTTC |
| *O. venulosum* | 280bp | 58 | 35 | This study | OvF: TGTTTACTACAGTGTGGCTTG |
|  |  |  |  |  | OvR: CGGTTGTCTCATTTCACAGGC |
| *H. contortus* | 321bp | 50 | 40 | Redman *et al*., 2008 | HcF: GTTACAATTTCATAACATCACGT |
|  |  |  |  |  | HcR: TTTACAGTTTGCAGAACTTA |
| *T. vitrinus* | 100bp | 52 | 35 | Wimmer *et al*., 2004 | TvF: AGGAACATTAATGTCGTTACA |
|  |  |  |  |  | TvR: CTGTTTGTCGAATGGTTATTA |
| *T. axei* | 67bp | 52 | 35 | Wimmer *et al*., 2004 | TaF: AGGGATATTAATGTCGTTCA |
|  |  |  |  |  | TaR: TGATAATTCCCATTTTAGTTT |
| *C. ovinia* | 158bp | 52 | 35 | Wimmer *et al*., 2004 | CoF:CATGTGTGATCCTCGTACTAGATAAGA |
|  |  |  |  |  | CoR: ATGAACCGTACACCGTTGTCA |
