# Supplementary material for: The Emergence of Resistance to the Benzimidazole Anthlemintics in Parasitic Nematodes of Livestock Is Characterised by Multiple Independent Hard and Soft Selective Sweeps
Source: PLoS Negl Trop Dis. 2015 Feb 6;9(2):e0003494. doi: 10.1371/journal.pntd.0003494 (PMC4319741; doi:10.1371/journal.pntd.0003494)
Supplement: S2 Table — (DOC) [file pntd.0003494.s009.doc]

Supplementary Table S2 Summary of bioinformatic screen for microsatellites of *H. contortus* and *T. circumcincta* and their experimental screening against a range of genetic divergent isolates

|  | ***H. contortus*** | ***T. circumcincta*** | |
| --- | --- | --- | --- |
| Database summary | | | |
| Sequence database | Hc_contigs.012706 | TCIR_supercontigs.120209 | |
| Sequence Length (bp) | 93, 216, 526 | 44, 435, 691 | |
| No. of contigs | 58, 172 | 22, 287 | |
| Average size of contig (bp) | 1, 602 | 1, 994 | |
| Bioinformatic screen | | | |
| No. of microsatellites identified^a^ | 677 | | 556 |
| Loci with a repeat purity of >80%^b^ | 435 | | 461 |
| Loci with flanking sequence^c^ | 260 | | 320 |
| Experimental Screen | | | |
| Number of microsatellites screened^d^ | 81 | | 83 |
| Isolates used as screen^e^ | MHco3(ISE) | | MTci5 |
|  | MHco10(CAVR) | | NzWS |
|  | MHco4(WRS) | | FrMe |
|  | Hco(UK-12/10/07) | | ScSo210 |
| No. of loci dropped due to: | | | |
| Non-amplification^d^ | 55 [0.6790] | | 63 [0.7590] |
| >2 copies from an individual worm^f^ | 13 [0.1605] | | 13 [0.1566] |
| Lack of allele polymorphism ^g^ | 7 [0.0864] | | 1 [0.0120] |
| No. of loci to pass experimental screen ^h^ | 6 (0.0741) | | 6 (0.0723) |

^a^Tandem Repeat Finder software used for bioinformatic search for microsatellites. The most stringent alignment parameters were utilised (match, +2; mismatch, -7; gap, -7) to identify all potential loci with a repeat motif size of <10bp (repeat pattern size, 2-10bp) and a minimum alignment score >50. ^b^Microsatellites with a repeat purity of <80% are believed to be less stable and therefore removed from analysis.

^c^Microsatellites lacking sufficient flanking sequence to allow primer design removed from analysis.

^d^Experimental screening required the successful amplification from bulk worm templates and from 4 different individual worms of each of the genetically divergent strains used. If a primer pair failed to amplify, a second set were designed and tested.

^e^*H. contortus* strains used for experimental screen were: the reference genome strain MHco3(ISE), a UK field isolate, Hco(UK-12/10/07) and two isolates that are highly genetically divergent from MHco3(ISE) namely MHco4(WRS) (Pairwise F_ST_: 0.153) and MHco10(CAVR) (Pairwise F_ST_: 0.270) . *T. circumincta* strains were: the reference strain MTci5, a UK field isolate (ScSo210) and two field isolates previously found to be genetically divergent from MTci5, namely FrGa (Pairwise F_ST_: 0.123) and NzWS (Pairwise F_ST_: 0.045) .

^f^Thirteen candidate microsatellite loci for *H. contortus* and *T. circumcicnta* were discarded due to the amplification of >2 alleles per diploid worm.

^g^The level of polymorphism of the majority of loci that had reached this stage of the screening process was high with very few microsatellites being monomorphic; 7 *H. contortus* loci and 1 *T. circumcincta* loci were dropped from the screen because of their limited discriminatory value.

Numbers in square brackets represents the proprtion of loci dropped from the total number of microsatellites to enter the experimental screening process.

^h^A panel of six *H. contortus* loci and six *T. circumcincta* loci passed all stages of the experimental screen, representing approximately a 7% success rate for both species (proportion indicated in brackets). The final loci were evaluated by amplification of 30 individual worms from each of the genetically distinct isolates described above (data not shown).
