# Supplementary material for: The Emergence of Resistance to the Benzimidazole Anthlemintics in Parasitic Nematodes of Livestock Is Characterised by Multiple Independent Hard and Soft Selective Sweeps
Source: PLoS Negl Trop Dis. 2015 Feb 6;9(2):e0003494. doi: 10.1371/journal.pntd.0003494 (PMC4319741; doi:10.1371/journal.pntd.0003494)
Supplement: S3 Table — (DOC) [file pntd.0003494.s010.doc]

Supplementary Table S3: New microsatellites developed for population genetics analysis of *T. circumcincta* (Tc) and *H. contortus* (Hc)

| Microsatellite | Repeat sequence | T_A_ (˚C) | Allele range^a^ | Primer sequences (5' → 3') |
| --- | --- | --- | --- | --- |
| Tc13604^b^ | [CAGGTA]_12_ TAGGTA CAGGCA CAG | 50 | 250-386 | F: CGATAAATGGTATTATCTG |
|  |  |  |  | R: GCTGCTATTAGAGGATAT |
| Tc2467^b^ | [TTTA]_15_ TCTA TT | 54 | 149-224 | F: AACGCTTTGAACCGTGTCGG |
|  |  |  |  | R: GCTGCCACATCAGCTTAGA |
| Tc2066^b^ | [GGCGAGTA]_9_ GGCGATTA GGCGAGAA GGCGTGTA GGTGAGTA GGCGAG | 50 | 189-309 | F: GAGCAACGACTGAACCTCAC |
|  |  |  |  | R: GCTGGAAGCATATTCTGCGC |
| Tc22274^b^ | [TGTA]_17_ TGTC TGAA TGT | 54 | 189-329 | F: ACAAAGTGCTCAAGTTAG |
|  |  |  |  | R: GGGGGTTCTATATACAGTA |
| Tc7989^b^ | GTCT]_17_ GTCC GTTT GTGT G [ | 50 | 133-232 | F: GATCTCACGTACTATGAA |
|  |  |  |  | R: CTATTGAATGTCGTACAG |
| Tc4504^b^ | [ACAT]_13_ ACAGAT ACAG ACA | 50 | 228-276 | F: TTATCACACCACTTCATT |
|  |  |  |  | R: GTCTTTAAACGCTAAATA |
| Hc12850^c^ | [ACAG]_42_ [ACAA]_2_ TCAG [GCAG]_2_ TCGG A | 54 | 263-311 | F: GATCTGAAGGAGCTAAGG |
|  |  |  |  | R: AGGGTAACTGTCTGAGAATC |
| Hc22193^c^ | [ACACAT]_10_ [ACACAC]_2_ [ATACAT]_2_ ATACAC ACATAC ACA | 54 | 203-221 | F: ATCCACTTTCACTCCTATATCA |
|  |  |  |  | R: GTGTGCGTGTATCTGTTG |
| Hc3086^c^ | [ACAG]_58_ AGAG GCAG TCAG ACGG ACA | 54 | 304-389 | F: AAGCCAACAAAAGACAAT |
|  |  |  |  | R: CACATATAGAGCACTTCTCTT |
| Hc53265^c^ | [TGT]_27_ TGC [TGG]_5_ [CGT]_8_ GGT T | 54 | 167-226 | F: TGTAGCTGGACTTACTTTAAATA |
|  |  |  |  | R: AGAAGTGGAAATGCTAGATG |
| Hc13507^c^ | [ATAATAAT]_14_ ATAATAGT AATAATAGT CATAATAAT A | 54 | 177-233 | F: TTTGGTAAAATAAACCTCTGTG |
|  |  |  |  | R: TCTCACCTTGTACTTTTCATTA |
| Hc2884^c^ | [CGAGCGAG]_6_ [CGACCGAC]_6_ CGAGC | 54 | 151-193 | F: TCGGCTGCTTTCATAGAC |
|  |  |  |  | R: GGTATCGACCAAGATTCAG |

T_A_ The optimal annealing temperature for each primer pair

^a^ Allele size range (bp) for a range of isolates *H. contortus*: Hco3(ISE), Hco4(WRS), Hco10(CAVR) and Hco(UK-12/10/07); *T. circumcinta*: MTci5, NzWS, FrGa and ScSo210

^b^ *T. circumcincta* loci

^c^ *H. contortus* loci
