# Supplementary material for: The Emergence of Resistance to the Benzimidazole Anthlemintics in Parasitic Nematodes of Livestock Is Characterised by Multiple Independent Hard and Soft Selective Sweeps
Source: PLoS Negl Trop Dis. 2015 Feb 6;9(2):e0003494. doi: 10.1371/journal.pntd.0003494 (PMC4319741; doi:10.1371/journal.pntd.0003494)
Supplement: S4 Table — (DOCX) [file pntd.0003494.s011.docx]

Supplementary Table S4 Population genetic data for the seven UK populations of *H. contortus* based on panel of 10 microsatellite loci

|  |  |  |  |  |  |  |  |  |  |  |  |
| --- | --- | --- | --- | --- | --- | --- | --- | --- | --- | --- | --- |
|  | Hc53265 | Hc2884 | Hc3086 | Hcms25 | Hc22c03 | Hc22193 | Hc12850 | Hc13507 | Hcms36 | Hcms40 | All loci |
| A_Tot_ | 9 | 23 | 21 | 22 | 6 | 9 | 20 | 13 | 4 | 6 |  |
| Hc37 (32^a^) | | | | | | | | | | | |
| N_o_ | 0 | 7 | 1 | 2 | 1 | 2 | 5 | 5 | 0 | 5 |  |
| H_e_ | 0.6091 | 0.7902 | 0.9281 | 0.8232 | 0.3147 | 0.5158 | 0.8155 | 0.7918 | 0.5531 | 0.4983 | 0.6640 |
| H_o_ | 0.2500 | 0.1600 | 0.2581 | 0.5667 | 0.2903 | 0.2333 | 0.3333 | 0.1111 | 0.5625 | 0.1852 | 0.2951 |
| p-value | 0 | 0 | 0 | 0.0022 | 0.4610 | 0.0001 | 0 | 0 | 0.9099 | 0.0004 |  |
| F_IS_ | 0.5934 | 0.8008 | 0.7252 | 0.3153 | 0.0785 | 0.55188 | 0.5959 | 0.8620 | -0.0173 | 0.6328 | 0.5020 |
| A (A_U_) | 7(1) | 10 | 17(2) | 11 | 4 | 4(1) | 9 | 7 | 3 | 3 | 7.50^b^ |
| N_f_ | 0.2634 | 0.5319 | 0.3528 | 0.2427 | 0.2012 | 0.3194 | 0.4217 | 0.5103 | 0.0997 | 0.4311 |  |
| Hc95 (32^a^) | | | | | | | | | | | |
| N_o_ | 0 | 10 | 1 | 2 | 3 | 1 | 5 | 5 | 1 | 1 |  |
| H_e_ | 0.5164 | 0.7516 | 0.9133 | 0.8729 | 0.5123 | 0.5378 | 0.8973 | 0.7680 | 0.5875 | 0.5854 | 0.6943 |
| H_o_ | 0.5 | 0.1818 | 0.3548 | 0.5667 | 0.3214 | 0.3226 | 0.2963 | 0.4444 | 0.4194 | 0.4194 | 0.3827 |
| p-value | 0.3934 | 0 | 0 | 0.0003 | 0.0293 | 0.0212 | 0 | 0 | 0.1054 | 0.0552 |  |
| F_IS_ | 0.0322 | 0.7624 | 0.6154 | 0.3547 | 0.3769 | 0.4042 | 0.6740 | 0.4259 | 0.2896 | 0.2870 | 0.3810 |
| A (A_U_) | 4 | 9(1) | 15 | 11 | 5 | 4(2) | 13(1) | 9(1) | 4 | 4 | 7.80^b^ |
| N_f_ | 0.1131 | 0.5824 | 0.3007 | 0.2540 | 0.3480 | 0.2450 | 0.4501 | 0.3697 | 0.2144 | 0.2139 |  |
| Hc110 (30^a^) | | | | | | | | | | | |
| N_o_ | 0 | 4 | 1 | 2 | 4 | 1 | 8 | 3 | 0 | 3 |  |
| H_e_ | 0.6226 | 0.7979 | 0.9208 | 0.8338 | 0.5973 | 0.2196 | 0.8571 | 0.7554 | 0.6130 | 0.5926 | 0.6810 |
| H_o_ | 0.4667 | 0.3462 | 0.4138 | 0.7857 | 0.2308 | 0.1035 | 0.3333 | 0.3333 | 0.4333 | 0.4074 | 0.3854 |
| p-value | 0.1018 | 0 | 0 | 0.2101 | 0 | 0.0129 | 0 | 0 | 0.0085 | 0.1402 |  |
| F_IS_ | 0.2537 | 0.5710 | 0.5550 | 0.0586 | 0.6183 | 0.5333 | 0.6170 | 0.5634 | 0.2966 | 0.3166 | 0.3705 |
| A (A_U_) | 6 | 11(2) | 15 | 12(1) | 5 | 3(1) | 11(2) | 9(1) | 4 | 4 | 8.0^b^ |
| N_f_ | 0.1603 | 0.3966 | 0.2748 | 0.1628 | 0.4097 | 0.2659 | 0.5333 | 0.3663 | 0.1728 | 0.3034 |  |
| Hc54 (32^a^) | | | | | | | | | | | |
| N_o_ | 0 | 6 | 0 | 0 | 1 | 0 | 4 | 15 | 1 | 4 |  |
| H_e_ | 0.3567 | 0.7059 | 0.9142 | 0.8110 | 0.3152 | 0.4578 | 0.8636 | 0.5758 | 0.6171 | 0.6903 | 0.6308 |
| H_o_ | 0.2188 | 0 | 0.6250 | 0.5313 | 0.0968 | 0.25 | 0.3214 | 0.2353 | 0.4839 | 0.4286 | 0.3191 |
| p-value | 0.0004 | 0 | 0 | 0 | 0 | 0.0051 | 0 | 0.0006 | 0.2517 | 0.0027 |  |
| F_IS_ | 0.3905 | 1.0 | 0.3198 | 0.3486 | 0.6965 | 0.4579 | 0.6321 | 0.5988 | 0.2188 | 0.3834 | 0.4358 |
| A (A_U_) | 5 | 6(1) | 14 | 14(3) | 4 | 3(1) | 9 | 4 | 4 | 6(2) | 6.90^b^ |
| N_f_ | 0.1881 | 0.5642 | 0.1569 | 0.1889 | 0.2912 | 0.2081 | 0.4112 | 0.6619 | 0.1942 | 0.3353 |  |
| Hc86 (32^a^) | | | | | | | | | | | |
| N_o_ | 0 | 4 | 0 | 0 | 3 | 0 | 4 | 2 | 1 | 4 |  |
| H_e_ | 0.5734 | 0.7792 | 0.9271 | 0.8284 | 0.4586 | 0.5223 | 0.9013 | 0.7475 | 0.4955 | 0.5734 | 0.6807 |
| H_o_ | 0.4375 | 0.2143 | 0.8125 | 0.4375 | 0.3448 | 0.1875 | 0.2857 | 0.4000 | 0.5161 | 0.4643 | 0.4100 |
| p-value | 0.0867 | 0 | 0.0232 | 0 | 0.0468 | 0.0002 | 0 | 0 | 0.4904 | 0.0031 |  |
| F_IS_ | 0.2399 | 0.7286 | 0.1253 | 0.4759 | 0.2513 | 0.6447 | 0.6870 | 0.4691 | -0.0424 | 0.1931 | 0.3614 |
| A (A_U_) | 8(1) | 12(3) | 17(1) | 10(1) | 5(1) | 3(1) | 14 | 9(1) | 3 | 4 | 8.50^b^ |
| N_f_ | 0.1550 | 0.4418 | 0.0526 | 0.2418 | 0.2950 | 0.2663 | 0.4334 | 0.2990 | 0.1557 | 0.3027 |  |
| Hc102 (30^a^) | | | | | | | | | | | |
| N_o_ | 0 | 8 | 0 | 0 | 3 | 0 | 7 | 3 | 2 | 4 |  |
| H_e_ | 0.4469 | 0.7548 | 0.8921 | 0.8412 | 0.5220 | 0.2096 | 0.8522 | 0.7163 | 0.5864 | 0.6765 | 0.6498 |
| H_o_ | 0.1333 | 0.1818 | 0.6333 | 0.6333 | 0.2222 | 0.0333 | 0.2609 | 0.2222 | 0.5357 | 0.3846 | 0.3241 |
| p-value | 0 | 0 | 0 | 0 | 0 | 0.0006 | 0 | 0 | 0.3050 | 0.0013 |  |
| F_IS_ | 0.7052 | 0.7634 | 0.2936 | 0.2503 | 0.5790 | 0.8432 | 0.6986 | 0.6938 | 0.0878 | 0.4363 | 0.4392 |
| A (A_U_) | 5 | 9 | 13 | 10(1) | 4 | 2 | 9 | 6 | 4 | 3 | 6.50^b^ |
| N_f_ | 0.2717 | 0.5528 | 0.1439 | 0.1520 | 0.3677 | 0.2386 | 0.5121 | 0.4076 | 0.2149 | 0.3599 |  |
| Hc101 (32^a^) | | | | | | | | | | | |
| N_o_ | 0 | 11 | 1 | 2 | 3 | 0 | 8 | 7 | 1 | 1 |  |
| H_e_ | 0.4876 | 0.7936 | 0.8915 | 0.8197 | 0.5351 | 0.5262 | 0.9015 | 0.7615 | 0.5689 | 0.6085 | 0.6894 |
| H_o_ | 0.3226 | 0.0500 | 0.4667 | 0.4828 | 0.3214 | 0.1613 | 0.3044 | 0.2083 | 0.5667 | 0.4333 | 0.3317 |
| p-value | 0.0025 | 0 | 0 | 0 | 0.0127 | 0 | 0 | 0 | 1.0 | 0.0314 |  |
| F_IS_ | 0.3421 | 0.9385 | 0.4808 | 0.4154 | 0.4037 | 0.6970 | 0.6674 | 0.7307 | 0.0040 | 0.2914 | 0.4342 |
| A (A_U_) | 7 | 8(1) | 16 | 9 | 4 | 3 | 13(2) | 6 | 4 | 4 | 7.40^b^ |
| N_f_ | 0.1831 | 0.6552 | 0.2385 | 0.2815 | 0.3234 | 0.2838 | 0.5182 | 0.5151 | 0.1547 | 0.2162 |  |

A_Tot_, total number of alleles for each marker across all populations. N_o_, apparent null homozygotes, i.e. number of worms in the population which failed to give an amplification product for a particular marker; H_e_, expected heterozygosity; H_o_, observed heterozygosity; F_IS_, inbreeding coefficient; P-values indicate a significant deviation from Hardy–Weinberg equilibrium following bonferroni correction; A, number of alleles; A_U_, number of alleles unique to that population; N_f_, estimated null allele frequency.

^a^ Total number of individuals genotyped for each population is given in parenthesis under the population name.

^b^ Mean number of alleles in each population for eight markers.
