# Supplementary material for: The Emergence of Resistance to the Benzimidazole Anthlemintics in Parasitic Nematodes of Livestock Is Characterised by Multiple Independent Hard and Soft Selective Sweeps
Source: PLoS Negl Trop Dis. 2015 Feb 6;9(2):e0003494. doi: 10.1371/journal.pntd.0003494 (PMC4319741; doi:10.1371/journal.pntd.0003494)
Supplement: S5 Table — (DOCX) [file pntd.0003494.s012.docx]

Supplementary Table S5 Population genetic data for the seven UK populations of *T. circumcincta* based on 10 microsatellite loci

|  | Mtg15 | Mtg67 | Mtg68 | Mtg73 | Tc22274 | Tc7989 | Tc4504 | Tc2066 | Tc2467 | Tc13604 | All loci |
| --- | --- | --- | --- | --- | --- | --- | --- | --- | --- | --- | --- |
| A_Tot_ | 21 | 18 | 15 | 5 | 26 | 29 | 20 | 22 | 24 | 31 |  |
| Tc37 (32^a^) | | | | | | | | | | | |
| N_o_ | 11 | 2 | 3 | 2 | 7 | 6 | 12 | 5 | 4 | 3 |  |
| H_e_ | 0.8339 | 0.7486 | 0.8342 | 0.5339 | 0.9567 | 0.9404 | 0.9075 | 0.8274 | 0.8779 | 0.9329 | 0.8394 |
| H_o_ | 0.1429 | 0.7000 | 0.6897 | 0.2667 | 0.2000 | 0.4231 | 0.1053 | 0.3333 | 0.3929 | 0.4138 | 0.3668 |
| p-value | 0 | 0.0496 | 0.3993 | 0.0011 | 0 | 0 | 0 | 0 | 0 | 0 |  |
| F_IS_ | 0.8322 | 0.0660 | 0.1759 | 0.5048 | 0.7943 | 0.5550 | 0.8868 | 0.6017 | 0.5571 | 0.5608 | 0.4453 |
| A (A_U_) | 10 | 7 | 7 | 4 | 19(2) | 18(2) | 12(2) | 8 | 17(2) | 19(1) | 12.1^b^ |
| N_f_ | 0.6218 | 0.1750 | 0.2253 | 0.3084 | 0.5375 | 0.4282 | 0.6748 | 0.4236 | 0.3834 | 0.3611 |  |
| Tc95 (21^a^) | | | | | | | | | | | |
| N_o_ | 7 | 0 | 3 | 1 | 4 | 0 | 6 | 4 | 2 | 2 |  |
| H_e_ | 0.8942 | 0.8397 | 0.8524 | 0.3039 | 0.9109 | 0.9373 | 0.9149 | 0.9055 | 0.8919 | 0.9445 | 0.8395 |
| H_o_ | 0.3571 | 0.7619 | 0.6667 | 0.2500 | 0.5294 | 0.5714 | 0.1333 | 0.4118 | 0.3684 | 0.3684 | 0.3457 |
| p-value | 0 | 0.1346 | 0.1655 | 0.4778 | 0 | 0 | 0 | 0 | 0 | 0 |  |
| F_IS_ | 0.6096 | 0.0948 | 0.2229 | 0.1810 | 0.4263 | 0.3962 | 0.8586 | 0.5529 | 0.5936 | 0.6164 | 0.3774 |
| A (A_U_) | 11(1) | 8 | 8(1) | 3 | 11 | 17(4) | 11 | 11 | 14(1) | 17(1) | 11.1^b^ |
| N_f_ | 0.5576 | 0.1116 | 0.2950 | 0.2622 | 0.3942 | 0.2237 | 0.6028 | 0.4382 | 0.3830 | 0.3932 |  |
| Tc110 (32^a^) | | | | | | | | | | | |
| N_o_ | 9 | 0 | 0 | 2 | 9 | 0 | 12 | 5 | 13 | 9 |  |
| H_e_ | 0.8531 | 0.7391 | 0.8194 | 0.2446 | 0.9304 | 0.9390 | 0.9244 | 0.8400 | 0.9317 | 0.9082 | 0.8130 |
| H_o_ | 0.4348 | 0.5625 | 0.4688 | 0.2 | 0.4783 | 0.5313 | 0.0500 | 0.1111 | 0.3158 | 0.3044 | 0.3457 |
| p-value | 0 | 0.2727 | 0.0002 | 0.4248 | 0 | 0 | 0 | 0 | 0 | 0 |  |
| F_IS_ | 0.4960 | 0.2419 | 0.4319 | 0.1850 | 0.4916 | 0.4382 | 0.9472 | 0.8699 | 0.6672 | 0.6699 | 0.4573 |
| A (A_U_) | 10 | 8 | 10(2) | 4 | 15(1) | 17(3) | 13(2) | 13(5) | 14 | 14(1) | 11.8^b^ |
| N_f_ | 0.484 | 0.1500 | 0.2230 | 0.2706 | 0.4763 | 0.2323 | 0.6751 | 0.5180 | 0.6157 | 0.5342 |  |
| Tc54 (32^a^) | | | | | | | | | | | |
| N_o_ | 10 | 0 | 7 | 1 | 3 | 8 | 18 | 5 | 15 | 15 |  |
| H_e_ | 0.8721 | 0.6840 | 0.8245 | 0.2628 | 0.9232 | 0.8679 | 0.8942 | 0.8742 | 0.8324 | 0.9305 | 0.7966 |
| H_o_ | 0.5 | 0.5625 | 0.4000 | 0.2258 | 0.3793 | 0.2083 | 0.1429 | 0.2963 | 0.0588 | 0.1177 | 0.2892 |
| p-value | 0.0114 | 0.0725 | 0 | 0.4910 | 0 | 0 | 0 | 0 | 0 | 0 |  |
| F_IS_ | 0.4324 | 0.1800 | 0.5200 | 0.1429 | 0.5934 | 0.7639 | 0.8452 | 0.6653 | 0.9313 | 0.8769 | 0.6668 |
| A (A_U_) | 17(3) | 10(1) | 8(1) | 4 | 15 | 12 | 9 | 13(2) | 8 | 13 | 10.9^b^ |
| N_f_ | 0.4880 | 0.1331 | 0.4454 | 0.2152 | 0.3743 | 0.5435 | 0.7536 | 0.4464 | 0.7185 | 0.7077 |  |
| Tc86 (32^a^) | | | | | | | | | | | |
| N_o_ | 17 | 0 | 5 | 2 | 12 | 10 | 21 | 8 | 20 | 9 |  |
| H_e_ | 0.7885 | 0.7961 | 0.8616 | 0.4017 | 0.9115 | 0.9302 | 0.9004 | 0.8910 | 0.9094 | 0.9353 | 0.8326 |
| H_o_ | 0.1333 | 0.5938 | 0.2963 | 0.4333 | 0.3000 | 0.6364 | 0 | 0.3750 | 0.2500 | 0.1304 | 0.3149 |
| p-value | 0 | 0.0114 | 0 | 1 | 0 | 0.0005 | 0 | 0 | 0 | 0 |  |
| F_IS_ | 0.8358 | 0.2573 | 0.6604 | -0.0802 | 0.6766 | 0.3210 | 1.0 | 0.5843 | 0.7339 | 0.8632 | 0.3909 |
| A (A_U_) | 7 | 11(1) | 10 | 3 | 15 | 14 | 8 | 13(2) | 10 | 16(1) | 10.7^b^ |
| N_f_ | 0.7343 | 0.1538 | 0.4444 | 0.2123 | 0.5984 | 0.4481 | 0.831 | 0.4856 | 0.7702 | 0.5976 |  |
| Tc102 (30^a^) | | | | | | | | | | | |
| N_o_ | 8 | 0 | 2 | 0 | 6 | 6 | 17 | 10 | 16 | 11 |  |
| H_e_ | 0.8224 | 0.7915 | 0.8435 | 0.3938 | 0.9415 | 0.8945 | 0.8615 | 0.8321 | 0.8995 | 0.8947 | 0.8175 |
| H_o_ | 0.3636 | 0.6000 | 0.3214 | 0.3667 | 0.2500 | 0.5417 | 0.1539 | 0.15 | 0.2143 | 0.2105 | 0.3172 |
| p-value | 0 | 0.0203 | 0 | 0.2033 | 0 | 0 | 0 | 0 | 0 | 0 |  |
| F_IS_ | 0.5636 | 0.2451 | 0.6233 | 0.0700 | 0.7386 | 0.3996 | 0.8273 | 0.8235 | 0.7686 | 0.7696 | 0.4464 |
| A (A_U_) | 11 | 13 | 9 | 4 | 18(1) | 13(1) | 8(2) | 9(1) | 11 | 11 | 10.7^b^ |
| N_f_ | 0.4956 | 0.1518 | 0.3632 | 0.1366 | 0.5054 | 0.3874 | 0.7532 | 0.6137 | 0.7214 | 0.6198 |  |
| Tc101 (32^a^) | | | | | | | | | | | |
| N_o_ | 9 | 0 | 1 | 0 | 3 | 7 | 11 | 5 | 10 | 10 |  |
| H_e_ | 0.8686 | 0.8368 | 0.8139 | 0.5114 | 0.9425 | 0.8931 | 0.8084 | 0.7939 | 0.8880 | 0.8573 | 0.8214 |
| H_o_ | 0.1304 | 0.7188 | 0.5484 | 0.4688 | 0.4483 | 0.2000 | 0 | 0.2222 | 0.1818 | 0.1364 | 0.3055 |
| p-value | 0 | 0.2641 | 0 | 0.5256 | 0 | 0 | 0 | 0 | 0 | 0 |  |
| F_IS_ | 0.8527 | 0.1430 | 0.3298 | 0.0847 | 0.5288 | 0.7796 | 1.0 | 0.7239 | 0.7990 | 0.8441 | 0.5074 |
| A (A_U_) | 10 | 12(1) | 11 | 5(1) | 19(1) | 11 | 9 | 6 | 12(1) | 10(1) | 10.5^b^ |
| N_f_ | 0.5911 | 0.1099 | 0.2165 | 0.1236 | 0.3480 | 0.5295 | 0.6658 | 0.464 | 0.5943 | 0.6068 |  |

A_Tot_, total number of alleles for each marker across all populations. N_o_, apparent null homozygotes, i.e. number of worms in the population which failed to give an amplification product for a particular marker; H_e_, expected heterozygosity; H_o_, observed heterozygosity; F_IS_, inbreeding coefficient; P-values indicate a significant deviation from Hardy–Weinberg equilibrium following bonferroni correction; A, number of alleles; A_U_, number of alleles unique to that population; N_f_, estimated null allele frequency.

^a^ Total number of individuals genotyped for each population is given in parenthesis under the population name.

^b^ Mean number of alleles in each population for eight markers.
