# Supplementary material for: The Emergence of Resistance to the Benzimidazole Anthlemintics in Parasitic Nematodes of Livestock Is Characterised by Multiple Independent Hard and Soft Selective Sweeps
Source: PLoS Negl Trop Dis. 2015 Feb 6;9(2):e0003494. doi: 10.1371/journal.pntd.0003494 (PMC4319741; doi:10.1371/journal.pntd.0003494)
Supplement: S6 Table — (DOCX) [file pntd.0003494.s013.docx]

Supplementary Table S6 Nomenclature of isotype-1 β-tubulin alleles identified in this study with corresponding GenBank numbers.

| Species | Resistant/ Susceptible | P167 | P198 | P200 | Allele name | GenBank No. |
| --- | --- | --- | --- | --- | --- | --- |
| *H. contortus* | Susceptible | F | A | F | Hs1 | KF483605 |
| *H. contortus* | Susceptible | F | A | F | Hs2 | KF483606 |
| *H. contortus* | Susceptible | F | A | F | Hs3 | KF483607 |
| *H. contortus* | Susceptible | F | A | F | Hs4 | KF483608 |
| *H. contortus* | Susceptible | F | A | F | Hs5 | KF483609 |
| *H. contortus* | Susceptible | F | A | F | Hs6 | KF483610 |
| *H. contortus* | Susceptible | F | A | F | Hs7 | KF483611 |
| *H. contortus* | Susceptible | F | A | F | Hs8 | KF483612 |
| *H. contortus* | Susceptible | F | A | F | Hs9 | KF483613 |
| *H. contortus* | Susceptible | F | A | F | Hs10 | KF483614 |
| *H. contortus* | Resistant | F | A | Y | Hr1 | KF483600 |
| *H. contortus* | Resistant | F | A | Y | Hr2 | KF483601 |
| *H. contortus* | Resistant | F | A | Y | Hr3 | KF483602 |
| *H. contortus* | Resistant | Y | A | F | Hr4 | KF483603 |
| *H. contortus* | Resistant | Y | A | F | Hr5 | KF483604 |
| *T. circumcincta* | Susceptible | F | A | F | Ts1 | KF483640 |
| *T. circumcincta* | Susceptible | F | A | F | Ts2 | KF483641 |
| *T. circumcincta* | Susceptible | F | A | F | Ts3 | KF483642 |
| *T. circumcincta* | Susceptible | F | A | F | Ts4 | KF483643 |
| *T. circumcincta* | Susceptible | F | A | F | Ts5 | KF483644 |
| *T. circumcincta* | Susceptible | F | A | F | Ts6 | KF483645 |
| *T. circumcincta* | Susceptible | F | A | F | Ts7 | KF483646 |
| *T. circumcincta* | Susceptible | F | A | F | Ts8 | KF483648 |
| *T. circumcincta* | Susceptible | F | A | F | Ts9 | KF483651 |
| *T. circumcincta* | Susceptible | F | A | F | Ts10 | KF483652 |
| *T. circumcincta* | Susceptible | F | A | F | Ts11 | KF483653 |
| *T. circumcincta* | Susceptible | F | A | F | Ts12 | KF483654 |
| *T. circumcincta* | Susceptible | F | A | F | Ts13 | KF483655 |
| *T. circumcincta* | Susceptible | F | A | F | Ts14 | KF483656 |
| *T. circumcincta* | Susceptible | F | A | F | Ts15 | KF483657 |
| *T. circumcincta* | Resistant | F | A | Y | Tr1 | KF483615 |
| *T. circumcincta* | Resistant | F | A | Y | Tr2 | KF483616 |
| *T. circumcincta* | Resistant | F | A | Y | Tr3 | KF483617 |
| *T. circumcincta* | Resistant | F | A | Y | Tr4 | KF483618 |
| *T. circumcincta* | Resistant | F | A | Y | Tr5 | KF483619 |
| *T. circumcincta* | Resistant | F | A | Y | Tr6 | KF483620 |
| *T. circumcincta* | Resistant | F | A | Y | Tr7 | KF483621 |
| *T. circumcincta* | Resistant | F | A | Y | Tr8 | KF483622 |
| *T. circumcincta* | Resistant | F | A | Y | Tr9 | KF483623 |
| *T. circumcincta* | Resistant | F | A | Y | Tr10 | KF483624 |
| *T. circumcincta* | Resistant | F | A | Y | Tr11 | KF483625 |
| *T. circumcincta* | Resistant | F | A | Y | Tr12 | KF483626 |
| *T. circumcincta* | Resistant | F | A | Y | Tr13 | KF483627 |
| *T. circumcincta* | Resistant | F | A | Y | Tr14 | KF483628 |
| *T. circumcincta* | Resistant | F | A | Y | Tr15 | KF483629 |
| *T. circumcincta* | Resistant | F | A | Y | Tr16 | KF483630 |
| *T. circumcincta* | Resistant | F | A | Y | Tr17 | KF483631 |
| *T. circumcincta* | Resistant | F | A | Y | Tr18 | KF483632 |
| *T. circumcincta* | Resistant | F | A | Y | Tr19 | KF483633 |
| *T. circumcincta* | Resistant | F | A | Y | Tr20 | KF483634 |
| *T. circumcincta* | Resistant | F | A | Y | Tr21 | KF483635 |
| *T. circumcincta* | Resistant | F | A | Y | Tr22 | KF483636 |
| *T. circumcincta* | Resistant | F | A | Y | Tr23 | KF483637 |
| *T. circumcincta* | Resistant | F | A | Y | Tr24 | KF483638 |
| *T. circumcincta* | Resistant | F | L | F | Tr25 | KF483639 |
| *T. circumcincta* | Resistant | F | L | F | Tr26 | KF483647 |
| *T. circumcincta* | Resistant | F | L | F | Tr27 | KF483649 |
| *T. circumcincta* | Resistant | F | L | F | Tr28 | KF483650 |
